# Motional timescale predictions by molecular dynamics simulations: Case study using proline and hydroxyproline sidechain dynamics

**DOI:** 10.1002/prot.24350

**Published:** 2014-09-17

**Authors:** Abil E Aliev, Martin Kulke, Harmeet S Khaneja, Vijay Chudasama, Tom D Sheppard, Rachel M Lanigan

**Affiliations:** Department of Chemistry, University College LondonLondon, WC1H 0AJ, United Kingdom

**Keywords:** NMR, molecular dynamics simulations, biomolecular force field, structure, conformational analysis, peptides, proteins, proline

## Abstract

We propose a new approach for force field optimizations which aims at reproducing dynamics characteristics using biomolecular MD simulations, in addition to improved prediction of motionally averaged structural properties available from experiment. As the source of experimental data for dynamics fittings, we use ^13^C NMR spin-lattice relaxation times *T*_1_ of backbone and sidechain carbons, which allow to determine correlation times of both overall molecular and intramolecular motions. For structural fittings, we use motionally averaged experimental values of NMR *J* couplings. The proline residue and its derivative 4-hydroxyproline with relatively simple cyclic structure and sidechain dynamics were chosen for the assessment of the new approach in this work. Initially, grid search and simplexed MD simulations identified large number of parameter sets which fit equally well experimental *J* couplings. Using the Arrhenius-type relationship between the force constant and the correlation time, the available MD data for a series of parameter sets were analyzed to predict the value of the force constant that best reproduces experimental timescale of the sidechain dynamics. Verification of the new force-field (termed as AMBER99SB-ILDNP) against NMR *J* couplings and correlation times showed consistent and significant improvements compared to the original force field in reproducing both structural and dynamics properties. The results suggest that matching experimental timescales of motions together with motionally averaged characteristics is the valid approach for force field parameter optimization. Such a comprehensive approach is not restricted to cyclic residues and can be extended to other amino acid residues, as well as to the backbone. Proteins 2014; 82:195–215. © 2013 Wiley Periodicals, Inc.

## INTRODUCTION

Molecular dynamics (MD) simulations are widely employed for structural and dynamics characterizations of peptides and proteins.^1^–^4^ These simulations rely mainly on classical force field parameters, such as AMBER,^5^–^7^ CHARMM,^8^ GROMOS,^9^,^10^ and OPLS-AA.^11^,^12^ Amongst different types of force field parameters, backbone, and sidechain torsional potentials have been the subject of extensive reoptimizations, leading to improved modifications of AMBER^13^–^16^ and CHARMM^17^,^18^ force fields. Based on a number of detailed benchmark studies,^19^–^33^ AMBER99SB^14^ has emerged as one of the force fields which reproduces experimentally measured parameters with better accuracy compared to other force fields. This force field has undergone further useful refinements in recent years.^15^,^16^ To predict the correct balance of secondary structure propensities in proteins, a simple backbone energy correction was introduced to reproduce the fraction of helix measured in short peptides at 300 K, with the modified force field known as AMBER99SB*.^15^ Recently, the AMBER99SB force field has been improved further (known as AMBER99SB-ILDN)^16^ by refitting the amino acid sidechain torsion potentials of the AMBER99SB force field for four residues: isoleucine, leucine, aspartic acid, and asparagine.

**Figure 1:**
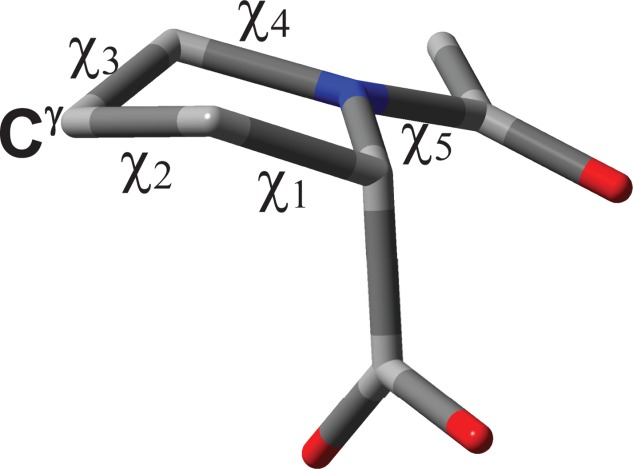
Diagram of NAcPro showing atom and dihedral angle labelling used. The C^γ^-endo conformer is shown. [Color figure can be viewed in the online issue, which is available at http://wileyonlinelibrary.com.]

One of the important properties not exploited in the force field optimizations for biomolecular MD simulations is the timescale of motion for a given backbone or sidechain fragment. As a result, while the motionally averaged experimental NMR parameters can be reproduced well by new force fields, the timescale over which this averaging is achieved may deviate significantly from experiment. The reason for the lack of timescale verifications is that either experimental data is not available or it is not clear how the force field parameters can be modified to reproduce better the experimental data. To explore the possibilities that involve experimentally known motional timescales in force field optimizations, we have selected a relatively simple example of the proline (Pro) sidechain dynamics in this work. The simplicity of the Pro dynamics arises from the fact that unlike other amino acid residues the Pro residue has a unique cyclic structure, which interconverts continuously between two conformers, known as C^γ^-endo and C^γ^-exo.^34^ Another factor in favor of the Pro residue is that numerous theoretical and experimental studies have been undertaken in the past focusing mainly on the pyrrolidine ring dynamics. Furthermore, the torsional parameters of the Pro residue have not been optimized in the past and standard force field parameters obtained for open chain fragments are used for proline. The result is that the predicted geometry of the pyrrolidine ring by AMBER force fields is relatively flat compared to single-crystal X-ray diffraction data or quantum-mechanical (QM) calculations, as judged by the value of the endocyclic torsion *χ*_2_ (Fig. 1) or the pseudorotation amplitude *χ*_m_, also known as the maximum puckering angle.^34^ In the first approximation, the nonplanarity of the pyrrolidine ring can be assessed by how far atoms C^β^ and C^γ^ are placed from the plane formed by the remaining three atoms. The further they are from the plane, the higher the absolute values of *χ*_2_ and *χ*_m_ are. We note that changes in geometry of the ring have also further energetic implications, and, as shown previously, the larger the maximum puckering angle the larger the pyrrolidine ring interconversion barrier in Pro and hydroxyproline (Hyp) residues.^35^ The increase in the energy barrier implies less frequent transitions or longer motional timescales. Based on these considerations, force field optimizations may potentially improve the accuracy of MD simulations for predicting both the structure and dynamics of the Pro residue in proteins. Note that one of the important attributes of the Pro residue is its hinge-like function, which enhances the probability of β-turns in proteins. Therefore, accurate predictions of the proline structure and dynamics may have critical implication on the outcome of MD descriptions of proteins.

Returning to the original problem of force field optimizations, we expect that the introduction of an additional dynamics constraint into force field optimizations should be advantageous from a methodological point of view, as multiple solutions are often found in force field optimizations which fit equally well experimental data. This is not surprising, as the experimental data consists of motionally averaged values of NMR *J* couplings and chemical shifts, which are dependent on the relative populations of conformers, but not on how fast they exchange. Timescale fittings combined with fittings of NMR *J* couplings and/or chemical shifts are expected to select a correct solution in such cases. Unlike previous optimizations based on the quantum-mechanical calculations, we will use experimentally measured NMR *J*-couplings in our initial re-optimization of the Pro sidechain torsion potentials. The approach used by us is based on either simple grid search or iterative fittings of experimental NMR data, in which a figure-of-merit function is evaluated using MD trajectories calculated for each trial set of parameters. Once torsional force fields reproducing experimental NMR *J*-couplings have been identified, we will probe MD-predicted timescales of motions which best match experimental data. ^13^C NMR spin-lattice relaxation times will be used to estimate both overall and intramolecular timescales of motions. In addition to the Pro residue, we will also reoptimize torsional force field parameters for the *trans*-4-hydroxy-L-proline residue (Hyp) to match experimental dynamics data.

## MATERIALS AND METHODS

### NMR Data

Apart from Ace-Hyp-NHMe (AHM) and Ace-Hyp-Gly (AHG), all other peptides were used as received from Sigma Aldrich and Cambridge Bioscience. The synthesis of AHM and AHG is described in Supporting Infromation. Experimental values of proton ^3^*J*_HH_ couplings and internuclear proton distances for *N*-acetyl-l-proline (NAcPro), Gly-Pro-Gly-Gly (GPGG) and Val-Ala-Pro-Gly (VAPG) in D_2_O solutions at 298 K were taken from Refs.^34^–^36^. The experimental data for angiotensin II, AHM and AHG was determined in this work (see below) using full lineshape analysis.^34^ Unless otherwise specified, the *trans*-orientation about the amide bond preceding the Pro (or Hyp) residue is assumed for a given peptide. For the values of ^3^*J*-couplings determined from the full lineshape analysis, the standard deviation was estimated to be <0.1 Hz.^34^–^36^ Experimental values of ^3^*J*-couplings for ubiquitin were taken from Refs.^37^ and^38^. The root-mean-square (rms) deviations in the 3D-derived ^3^*J* values of ubiquitin were estimated to be of the order of ∼0.1 Hz.^37^

Solution ^1^H NMR spectra were recorded on a Bruker Avance III 600 MHz NMR spectrometer equipped with a 5 mm cryoprobe (^1^H 600.13 MHz and ^13^C 150.90 MHz). Data acquisition and processing were performed using standard TopSpin (version 2.1) software. ^1^H and ^13^C chemical shifts were calibrated using dioxane shifts in D_2_O (^1^H 3.75 ppm, ^13^C 67.19 ppm). Uncertainties in measured values of ^1^H and ^13^C chemical shifts were typically better than ±0.01 ppm. Unless otherwise specified, NMR measurements were carried out at 298 K. High (>300 K) and low (<300 K) temperature calibrations were carried out using standard samples of 80% 1,2-ethanediol in DMSO-*d*_6_ and 4% CH_3_OH in CD_3_OD, respectively.

The ^13^C spin-lattice relaxation times were measured for solutions of peptides in either D_2_O or H_2_O:D_2_O (9:1) using a standard inversion-recovery technique with the ^13^C observation in the presence of proton decoupling. To minimize errors associated with low signal-to-noise ratios, these experiments were carried out on a 600 spectrometer with a dual channel ^1^H/^13^C cryoprobe with the sensitivity optimised for ^13^C measurements. From five independent measurements carried out at probe ambient temperature (293 K) for the 214 m*M* solution of GPGG in D_2_O at different dates over 60 days, the standard deviations for ^13^C *T*_1_ measurements were within 0.4–1.4% of the corresponding mean values. From three independent measurements carried out at 298 K for the 77 m*M* VAPG in H_2_O:D_2_O (9:1), the standard deviations for ^13^C *T*_1_ measurements were within 0.2–1.1% of the corresponding mean values.

Chemical shift anisotropies (Δσ, in ppm) of aliphatic carbons were measured using slow MAS measurements (2.5 kHz) on a Bruker AVANCE III 850 spectrometer equipped with a 4 mm CPMAS probe and a solid sample of L-proline. The estimated Δσ values (−43 ppm for C^α^ and −30 ppm for C^γ^ of L-proline) were used in calculations of correlation times using ^13^C *T*_1_ values. From the ^13^C *T*_1_ calculations, at Δσ = −43 ppm, the dipolar relaxation mechanism remains the dominant factor determining ^13^C *T*_1_ relaxation at 14.1 T, while chemical shift anisotropy accounts for <1% of *T*_1_ values.

### MD calculations and simplex fittings

All MD simulations were carried out using *GROMACS* (version 4.5.5).^39^ One molecule of NAcPro molecule (terminated with CO_2_^−^ and with a Na^+^ cation added for neutralization) was solvated with 147 water molecules in a dodecahedral box with a volume of 4.7 nm^3^ in MD simulations. Periodic boundary conditions and the TIP3P water model^40^ were employed in all MD simulations. An integration step of 2 fs was used and neighbor lists were updated every 5th step. The particle mesh Ewald (PME)^41^ method was employed for the electrostatics with fourth-order interpolation. The neighbor list and the real-space cutoff distances were set to 0.9 nm, which is similar to that used in optimizations of the original force field and its recent modifications.^5^–^7^,^13^–^16^ The van der Waals interactions in all MD simulations were treated with a twin-range cutoff method using the neighbor list and van der Waals cutoff distances. The value of the van der Waals cutoff distance was 0.9 nm.^5^–^7^,^13^–^16^ The temperature at 298 K was controlled using velocity rescaling with a stochastic term (V-rescale)^42^ and a time constant of 0.1 ps. A Parrinello–Rahman scheme was employed for pressure control at 1 bar using a coupling constant of 2 ps and an isothermal compressibility of 4.5 × 10^-5^ bar^−1^.^43^ Prior to production MD runs, including those implemented within downhill simplex optimizations,^44^,^45^ the system was minimized using steepest-descent and conjugate gradient algorithms. Minimization steps were followed by four steps of equilibration. The system was equilibrated for 40 ps with the positionally restrained solute molecule to allow water molecules to equilibrate around it, followed by a *NVT* molecular dynamics for 100 ps, *NPT* dynamics for 200 ps and another *NVT* dynamics for 200 ps. Reproducible production MD simulations at each step of simplex fittings were performed for 7.5–40.5 ns using *NVT* ensemble, the first 0.5 ns of which was discarded from the calculations of averaged NMR parameters. For the selected set of parameters from simplex fittings additional 200 ns long MD simulations were carried out.

The vicinal ^3^*J* couplings of the five-membered pyrrolidine ring in NAcPro **(**as well as in other peptides, see below) in each frame of MD simulations were calculated using empirically optimized Karplus-type equations 8C and 8D of Haasnoot *et al*.^46^ These equations contain terms accounting for the differences in electronegativities of α- and β-substituents, and hence are better suited for the analysis of the ^3^*J* couplings of the pyrrolidine ring than the original Karplus equation.^47^ The precision of equation 8C of Haasnoot *et al*. (expressed as the rms deviation) for a structural fragment containing 2 substituents (-CH_2_X-CH_2_Y-) is estimated as 0.367 Hz using a set of 45 experimental ^3^*J*_HH_ couplings.^46^ The precision of equation 8D of Haasnoot *et al*. for a structural fragment containing 3 substituents (-CHXY-CH_2_Z-) is estimated as 0.485 Hz using a set of 100 experimental ^3^*J*_HH_ couplings.^46^

To analyze MD trajectories, including those obtained at each step of simplex fittings, dihedral angles were extracted for each frame recorded every 0.01 ps during the MD simulation. The calculated values of ^3^*J* couplings using the corresponding dihedral angles in each frame were used to calculate the averaged values of ^3^*J* couplings over the duration of the MD simulation. The rms deviation defined as
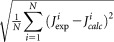
 (denoted as rms_*Jp*_ for the ^3^*J*_HH_-couplings of the pyrrolidine ring) was used as a figure-of-merit function in simplex fittings, where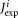 and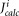 are conformationally averaged experimental and calculated couplings, respectively, and *N* is the number of different *J* couplings available (*N* = 10 for the Pro sidechain). As simplex may in principle lead to a local minimum of the merit function,^44^,^45^ it is important to consider several sets of starting values of the optimized parameters *x*_j_. This was achieved by varying the factor *c*, by which one of the optimized parameters *x*_j_ is varied within the first *n* + 1 steps of the simplex run using the following expression: *x*_j_ + *c x*_j_ (i.e., at step *n* = 1 the initial values of *x*_j_ from the original AMBER99SB force field are used followed by *x*_1_ +*c x*_1_, *x*_2_, … *x*_n_ at step *n* = 2, etc.). Several simplex fittings were considered with *c* varied between 0.2 and 5 (see the main text for further details). In addition, for |*c*| < 1, both positive and negative values were considered. An additional constraint requiring *x*_j_ > 0 was imposed in simplex fittings.

For further optimization and validation of newly derived force field parameters, 800 ns MD simulations of GPGG, VAPG, Gly-Pro-Phe (GPF), 1.5 μs MD simulations of angiotensin II, 1 μs MD simulations of human ubiquitin (PDB entry 1UBQ),^48^ 600 ns and 1.5 μs MD simulations of AHM and 1.5 μs MD simulations of AHG were carried out. One molecule of zwitterionic GPGG was solvated with 253 water molecules in a dodecahedral box with a volume of 8.3 nm^3^. For VAPG, one molecule of zwitterionic peptide was solvated with 260 water molecules in a dodecahedral box with a volume of 8.4 nm^3^. In the case of GPF, one molecule of zwitterionic peptide was solvated with 292 water molecules in a dodecahedral box with a volume of 9.3 nm^3^. Similarly, one molecule of angiotensin II (with a Cl^-^ anion added for neutralization) was solvated with 1201 water molecules in a dodecahedral box with a volume of 40.8 nm^3^. One molecule of ubiquitin (with six Na^+^ cations added for neutralization) was solvated with 2605 water molecules in a cubic box with a volume of 91.1 nm^3^. For the Hyp parameter optimizations, one molecule of AHM was solvated with 225 water molecules in a dodecahedral box with a volume of 7.4 nm^3^ and one molecule of AHG (with a Na^+^ cation added for neutralization) was solvated with 300 water molecules in a dodecahedral box with a volume of 9.4 nm^3^. Other conditions and parameters of MD simulations were the same as described above for NAcPro. Frames recorded every 1 ps were used in estimating averaged ^3^*J*-couplings from MD simulations of GPGG and ubiquitin.

The calculated ^3^*J*_HH_ couplings are expected to depend on the length of the MD simulation. To estimate the significance of this dependence, we have considered MD simulations of varying lengths. Calculations of ^3^*J*_HH_ couplings using 600, 700, and 800 ns long MD simulations of GPGG using the modified force field (referred to as (25), Table1) showed the largest variation of less than ±0.023 Hz in the calculated ^3^*J*_HH_ values over 200 ns change in the length of the MD simulation (<0.5% of the value of the ^3^*J*_HH_ coupling). Two MD simulation of GPGG with 800 ns and 3 μs lengths were available for the parameter set (19), with the third largest value of *V*_3_ considered (6.92437 kJ mol^−1^, Table1). These were used for error estimates in MD-predicted quantities. The changes were (see Tables4 for definitions of parameters): *P*_exo_ 0°, *P*_endo_ 0°, χ_m_ +0.1°, *x*_endo_ +0.9%, rms_*Jp*_ +0.025 Hz, *p*_f_ 0 %, *d*_ter_ 0 Å, *N*^ψ1^ +0.41, *N*^ψ2^ +0.04, *N*^ϕ3^−0.33, *N*^ψ3^ −0.29, *N*^χ1^ −0.02, *N*^χ2^ +0.01, *S*^2^ 0, τ_*i*_ −0.02 ps. The negative sign here corresponds to the decrease of the value on increasing the length of the MD simulation. The absolute values of these changes can be considered as an estimate of the upper limit of errors involved, as the value of the force constant in parameter set (19) is higher than that in the final selected set (25), hence requiring longer MD simulations for better convergence in calculated parameters in the case of (19).

**Table 1:** Summary of Torsional Force Constants (*V*_n_, in kJ mol^−1^), Their Phases (γ_n_, in Degrees) and the Pyrrolidine Ring Conformational Characteristics of NAcPro^a^

	*V*_1_ (kJ mol^−1^)	*V*_2_ (kJ mol^−1^)	*V*_3_ (kJ mol^−1^)	*V*_4_ (kJ mol^−1^)	*V*_5_ (kJ mol^−1^)	*V*_6_ (kJ mol^−1^)	*P*_exo_(°)	*P*_endo_ (°)	*χ*_m_ (°)	*x*_endo_ (%)	rms_*Jp*_ (Hz)
AMBER99SB	0.8368	1.046	0.75312	0	0	0	14	178	35.5	56.7	0.935
1	1.02821	0.85218	1.37935	0	0	0	14	178	35.8	56.3	0.893
2	0.35073	0.32171	1.29055	0	0	0	14	178	36.5	56.3	0.866
3	0.8368	0.58111	1.75728	0	0	0	14	178	36.2	55.8	0.879
4	0.13985	0.20968	1.06169	0	0	0	14	178	36.0	57.0	0.854
5	0.16736	0.20920	1.95811	0	0	0	13	179	37.0	56.4	0.807
6	0	0	9.31503	0	0	0	11	180	41.2	46.2	1.402
7	0	0	6.61951	0	0	0	11	180	40.0	56.4	0.738
8	0	0	2.25938	0	0	0	13	179	37.0	56.4	0.802
9	0	0	5.51626	0	0	0	12	180	39.2	55.3	0.792
10	0	0	4.17167	0	0	0	12	179	38.5	56.5	0.742
11	0	0	3.30976	0	0	0	12	179	38.0	54.7	0.851
12	0	0	3.58557	0	0	0	13	179	38.0	54.9	0.833
13	0	0	3.79243	0	0	0	12	179	38.0	57.0	0.728
14	0	0	2.3954	0	0	0	13	179	37.3	55.7	0.828
15	0	0	2.6885	0	0	0	13	179	37.3	55.5	0.825
16	0	0	3.028	0	0	0	13	179	37.8	55.3	0.828
17	0	0	6.35714	0	0	0	12	180	40.0	55.0	0.808
18	0	0	7.17114	0	0	0	11	180	39.5	51.2	1.044
19	0	0	6.92437	0	0	0	11	180	40.0	50.6	1.078
20	0	0	4.42712	0	0	0	12	179	38.7	53.5	0.899
21	0	0	4.81624	0	0	0	12	180	38.5	55.5	0.786
22	0	0	4.6633	0	0	0	12	180	38.3	55.0	0.810
23	0	0	4.06	0	0	0	12	179	38.2	55.0	0.817
24	0	0	0	4.0284	2.82	0.5662	13	179	37.5	53.4	0.899
25	0	0	4.3474	0	0	0	13	179	38.3	55.1	0.814
NMR^34^	—	—	—	—	—		14	185	40.3	61.1	0.49^b^

a γ_1_ = γ_2_ = 180° and γ_3_ = γ_4_ = γ_5_ = γ_6_ = 0°.

b From least-squares fittings of the vicinal ^3^*J*-couplings^34^ using Eqs. (8C) and (8D) of Haasnoot *et al*.^46^

The motionally averaged ^3^*J*-couplings of the peptide backbone of GPGG and ubiquitin were calculated using quantum-mechanically derived Karplus relationships^31^,^49^ and empirically parameterized Karplus equations.^50^,^51^

Interatomic distances from the MD simulations of GPGG were calculated in a manner similar to that used in NMR measurements^36^: (i) internuclear distances (*r_i_*) for pairs of hydrogen atoms were calculated in each MD frame *i*; (ii) a quantity equal to *r*^−6^ was calculated as a measure of the expected NOE in each frame, *η_i_*; (iii) the sum of *r_i_*^−6^ were used as a measure of the expected total NOE over the full length of the MD run; (iv) using *r* = 2.4 Å as the reference H^α^-H^β3^ distance in the Pro residue,^36^ internuclear distances for other proton pairs were calculated using the *η* ∼*r*^−6^ relationship.

As shown by Tropp,^52^ when overall molecular motions are relatively slow and intramolecular motions are relatively fast, NOEs may show a *r*^−3^ dependence, for example, in globular macromolecules. In the case of the tetrapeptide GPGG used in this work for the NOE analysis, timescales of overall and intramolecular motions are both relatively fast. We have therefore used the *r*^−6^ dependence of NOEs. This is consistent with the simplified growth rates method used widely for interproton distance measurements in small molecules.^53^–^57^

To determine autocorrelation times for the intramolecular motions of the C-H bond from MD simulations, the corresponding internal autocorrelation functions were calculated using the following equation^58^: (1)
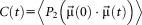
where 〈…〉 denotes an average over the MD trajectory,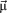 is a unit vector along the C—H bond direction and *P*_2_ is the second order Legendre polynomial. Prior to the *C*(*t*) calculations, the overall rotational and translational motions of the solute molecule were removed from the MD trajectory. This was accomplished by superimposing the sequence of four bonded peptide backbone atoms C(Pro)-C^α^(Pro)-N(Pro)-C(*i*+1) on the corresponding atoms of the snapshot at the midpoint of the production run, chosen as the reference structure. A similar approach was used by Showalter and Brüschweiler in their detailed analysis of NMR relaxation data (for a detailed discussion see Section 2.3 of Ref.^25^). The Lipari–Szabo model was used to fit the initial 20 ns of the autocorrelation*C*(*t*) functions^59^: (2)
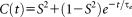


In Eq. (2) above, *S*^2^ denotes the order parameter and τ_*e*_ is the autocorrelation time for the intramolecular C—H bond reorientations.^59^

### Quantum-mechanical calculations

All quantum-mechanical calculations were carried out using *Gaussian 09*.^60^ Geometry optimizations were carried using various combinations of QM methods and basis sets, as described in the main text. The “nosymm” keyword of *Gaussian 09* was employed to carry out QM calculations with the symmetry of molecules disabled. For DFT M06-2X^61^,^62^ geometry optimizations, the ultrafine numerical integration grid (with 99 radial shells and 590 angular points per shell) was used, combined with the “verytight” convergence condition (requesting the root-mean-square forces to be smaller than 1 × 10^−6^ Hartree Bohr^−1^). Additional frequency calculations were also undertaken to verify that the optimized geometries correspond to true minima. The reaction field method IEFPCM^63^,^64^ was used to account for water solvent effects. The jump angles *Δθ* of the C—H bonds as a result of the pyrrolidine ring interconversion were determined using Python Molecular Viewer (version 1.5.4).^65^

Calculations employing MP2 and M06-2X methods were also carried out in which a selected dihedral angle was incremented or decremented in 5° steps. Basis sets considered are specified in the main text. At each step the selected dihedral angle was fixed with all the remaining degrees of freedom optimized using MP2 or M06-2X QM calculations. A relaxed 1D potential energy surface scan was performed in this manner and minimized QM energies at each step were obtained. The QM-optimized structures were then used in molecular mechanics (MM) calculations using AMBER99SB force field to obtain the corresponding MM energies (see the main text for further details).

### Conformational notation

The original conformational notation proposed by Haasnoot *et al*. for L-prolines are used in this work.^66^ The exo- and endo-orientations of the Pro ring carbon C^γ^ are defined relative to the substituent (COO or CONH groups) at the C^α^ carbon of the Pro ring. The definition of endo- and exocyclic torsional angles is shown in Figure 1.

The pseudorotation phase angle, *P*, which identifies a given conformation on the pseudorotation circle,^66^ and the pseudorotation amplitude *χ*_m_, which is the maximum value attained by χ_1_-χ_5_.^66^ The calculations of *P* and χ_m_ were done using equations by Westhof–Sundaralingam^67^: (3)
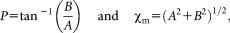
where (4)
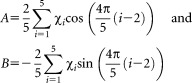


Note that 180° is added to the calculated value of *P* if *χ*_2_ < 0. From the distributions of endocyclic torsional angles, a two-site exchange between C^γ^-endo and C^γ^-exo conformations of the pyrrolidine ring of Pro and Hyp residues was observed in MD simulations of the peptides considered. The populations of these ring conformations are denoted as *x*^endo^ and *x*^exo^ (in % with *x*^endo^ + *x*^exo^ = 100%).

## RESULTS

### Initial simplexed MD fittings of experimental NMR data

In our initial revision of the AMBER99SB force field we undertook simplex fittings of ^3^*J*_HH_-couplings, which comprised the optimization of the C-C-C-C dihedral parameters for the endocyclic carbons in the Pro residue of *N*-acetyl-L-proline (NAcPro) and Gly-Pro-Gly-Gly (GPGG). The choice here is dictated by the fact that accurate experimental data is available for NAcPro and GPGG.^34^–^36^ In particular, full lineshape analysis was employed to derive accurate experimental values of ^3^*J*_HH_-couplings in D_2_O solutions, with the estimated standard deviation ≤0.03 Hz for vicinal couplings.^34^ As for the choice of the force field, the analysis of >10 different force fields applied to GPGG, identified AMBER99SB as the force field which reproduces best experimentally measured NMR parameters in aqueous solutions.^31^ Thus, further improvement of this force field presents a challenging task for the simplex fittings of ^3^*J*_HH_ couplings.

While AMBER99SB predicts satisfactorily the relative energies of C^γ^−exo and C^γ^−endo conformations (as judged by their populations predicted by AMBER99SB MD simulations and those determined experimentally from least squares fittings of ^3^*J*_HH_-couplings),^31^,^35^,^36^ the predicted number of χ_2_ transitions in the Pro-2 residue of GPGG is nearly four times higher than the number of the backbone ψ transitions of Gly-3 (see Table2 in Ref.^31^). This is in disagreement with the available experimental data. In particular, from the auto-correlation times and activation parameters reported for GPGG in water based on ^13^C spin-lattice relaxation time measurements,^68^ the frequency of the torsional transitions involving the C^γ^ atom of Pro-2 is of similar order of magnitude as the frequency of the torsional transitions involving the C^α^ atom of Gly-3 (see Tables1 and 2 in Ref.^68^). Thus, the Pro force field parameters must be optimized such that they reproduce experimentally observed timescale of the Pro sidechain motions. As discussed above, apart from dynamics aspects, there is also need for improving the predicted structure of the pyrrolidine ring. The geometry of the pyrrolidine ring as predicted by AMBER99SB MD simulations is flatter (*χ*_m_ ≈ 35°, where *χ*_m_ is approximately the same as the largest of the ring endocyclic torsions *χ*_1_−*χ*_5_, which is usually *χ*_2_) compared to NMR, X-ray and QM calculations (*χ*_m_ = 37°−42°).^34^,^35^ The reason for such a difference is that the same set of dihedral C—C—C—C parameters is used in AMBER force fields for both the cyclic (e.g., C^α^−C^β^−C^γ^−C^δ^ in Pro corresponding to the endocyclic torsion χ_2_) and open chain systems (see Ref.^69^ for details of how the C—C—C—C parameter was derived).

**Table 2:** Conformational Populations and Geometries of the Pro ring in GPGG in Water as Predicted by NMR and by 800-ns Long MD Simulations Using Various Sets of Torsional Parameters for the Pro residue

	*P*_exo_ (°)	*P*_endo_ (°)	*χ*_m_ (°)	*x*_endo_ (%)	rms_Jp_ (Hz)
AMBER99SB	14	180	35.3	58.9	0.662
1	14	181	36.5	59.0	0.618
2	13	180	36.4	59.2	0.601
3	14	180	36.7	59.1	0.588
4	13	180	36.4	59.2	0.611
5	13	181	37.7	59.3	0.561
6	11	182	41.3	56.3	0.562
7	12	181	40.0	59.3	0.562
8	13	181	37.0	58.9	0.544
9	12	182	39.3	57.3	0.486
10	12	181	38.7	58.9	0.520
11	13	181	38.3	58.7	0.517
12	12	181	38.3	58.6	0.511
13	12	181	38.4	58.2	0.499
14	13	181	37.6	59.1	0.547
15	12	180	37.8	59.1	0.540
16	13	181	37.9	58.4	0.513
17	11	182	39.8	58.3	0.523
18	12	181	40.2	59.3	0.572
19	11	181	40.0	57.9	0.524
20	12	181	38.8	59.1	0.526
21	12	181	39.1	59.2	0.530
22	12	181	39.0	59.0	0.525
23	12	181	38.5	58.4	0.502
24	13	180	37.7	57.7	0.483
25	12	181	38.7	59.2	0.529
NMR^35^	11	189	41	54.3	0.49^a^

a From least-squares fittings of the vicinal ^3^*J*-couplings^35^ using Eqs. (8C) and (8D) of Haasnoot *et al*.^46^

For our initial simplex optimizations, a standard AMBER dihedral energy term of the following form was used: (5)
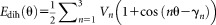
where *V_n_* represents dihedral force constant (amplitude), *n* is dihedral periodicity and γ_*n*_ with the value of either 0° or 180° is a phase of the dihedral angle θ. The dihedral force constants, *V_n_*, were optimized to obtain the best agreement between experimental and MD-predicted values of ^3^*J*-couplings of NAcPro. These are optimized for the angle *χ*_2_ (Fig. 1), which is usually the largest amongst the endocyclic dihedral angles *χ*_1_−*χ*_5_ for the Pro sidechain in peptides and proteins. There are three non-zero *V*_n_ values (*V*_1_, *V*_2_, and *V*_3_) for the *χ*_2_ = CT-CT-CT-CT torsion (CT denotes tetrahedral carbon) in the original AMBER99SB force field. Thus, three parameters *V*_1_, *V*_2_, and *V*_3_ were optimized in our simplex fittings, each step of which consisted of MD simulation followed by the calculation of the MD-averaged ^3^*J*_HH_ couplings using the modified Karplus equation of Haasnoot *et al*.^46^

Prior to deciding the length of MD simulation within simplex fittings, we examined the convergence of the population of endo conformation (*x*^endo^, in %) using a 500 ns long MD simulation (Fig. S1, Supporting Information). The results indicate that after the initial ∼10 ns the populations of the two conformers have converged sufficiently. In particular, after 10 ns MD run the population of the endo conformer is 56.6% compared to 56.3% after 20 ns, 56.5 after 100 ns and 56.7% after 500 ns. Even in the region between 1.5 and 10 ns, the population deviations are within less than ±2.0% (Fig. S1, Supporting Information). We have therefore used 7.5 ns long MD simulations at each step in our simplex fittings. The first 0.5 ns were considered as equilibration period and the corresponding data were discarded from calculations of averaged ^3^*J*_HH_-couplings. Up to 10 different simplexed MD simulations were carried out using different scaling factors *c* between −0.5 and 5, with 50–200 steps of 7.5 ns long MD simulations in each case.

The original AMBER99SB values of force field parameters, together with those derived from our simplex fittings of experimental ^3^*J*_HH_-couplings are shown in Table1. Five sets of optimized parameters (1)–(5) were selected from simplex fittings, showing the rms deviations from the experimental ^3^*J*_HH_-couplings (rms_*J*p_, in Hz) less than 0.8 Hz based on 7 ns long MD simulations. For comparison, rms_*J*p_ = 0.96 Hz for the original AMBER99SB force field. Considering that the increase in force constants during simplex optimizations may lead to longer convergence times, we used additional 200-ns long MD simulations for final estimates of merit functions (rms_*J*p_) for parameter sets (1)–(5) and AMBER99SB. The results of these simulations are summarized in Table1.

As can be seen from Table1, parameter sets (1)–(5) obtained from simplexed MD simulations show 5–14% improvements in rms values compared to the original AMBER99SB force field. The *χ*_m_ values in (1)–(5) have slightly increased compared to that in the original force field, which are in better agreement with the NMR, XRD and QM results (37–42).^34^,^35^ From the *E*_dih_(*χ*_2_) graphs for the CT-CT-CT-CT fragment (Fig. S2, Supporting Information), it can be seen that the *E*_dih_(*χ*_2_) graphs for the parameter sets (1)–(4) show higher maxima at *χ*_2_ = 0°, the values of which correspond to the value of *V*_3_, since *n* = 1 and *n* = 2 terms of Eq. (5) are zero at *χ*_2_ = 0°, as *γ*_1_ = *γ*_2_ = 180°. In the transition state between the C^γ^-endo and C^γ^-exo conformations of the pyrrolidine ring, the value of *χ*_2_ is 0°. Thus, the increase of the *V*_3_ value here corresponds to the increase of the activation energy of the ring interconversion. Based on the Arrhenius relationship, the increase of the activation energy is expected to lead to the decrease of the frequency of transitions between the C^γ^-endo and C^γ^-exo states.

The above results suggest that relatively short MD simulations combined with subsequent long MD simulations using selected sets can be applied for the refinement of force field parameters provided that the force constants do not increase significantly. Note that the simplex fittings described in this work generate a new MD trajectory for each trial set of parameters to evaluate the rms deviation between experimental and MD-predicted NMR data, that is, new conformations are created at each step of fittings (see Single Trajectory Reweighting Approach section below). However, the disadvantage of the current method is that it is computationally expensive and relatively large increase in optimized parameters may not be described adequately by short MD simulations used in simplex fittings.

### QM optimizations of force field parameters

**Figure 2:**
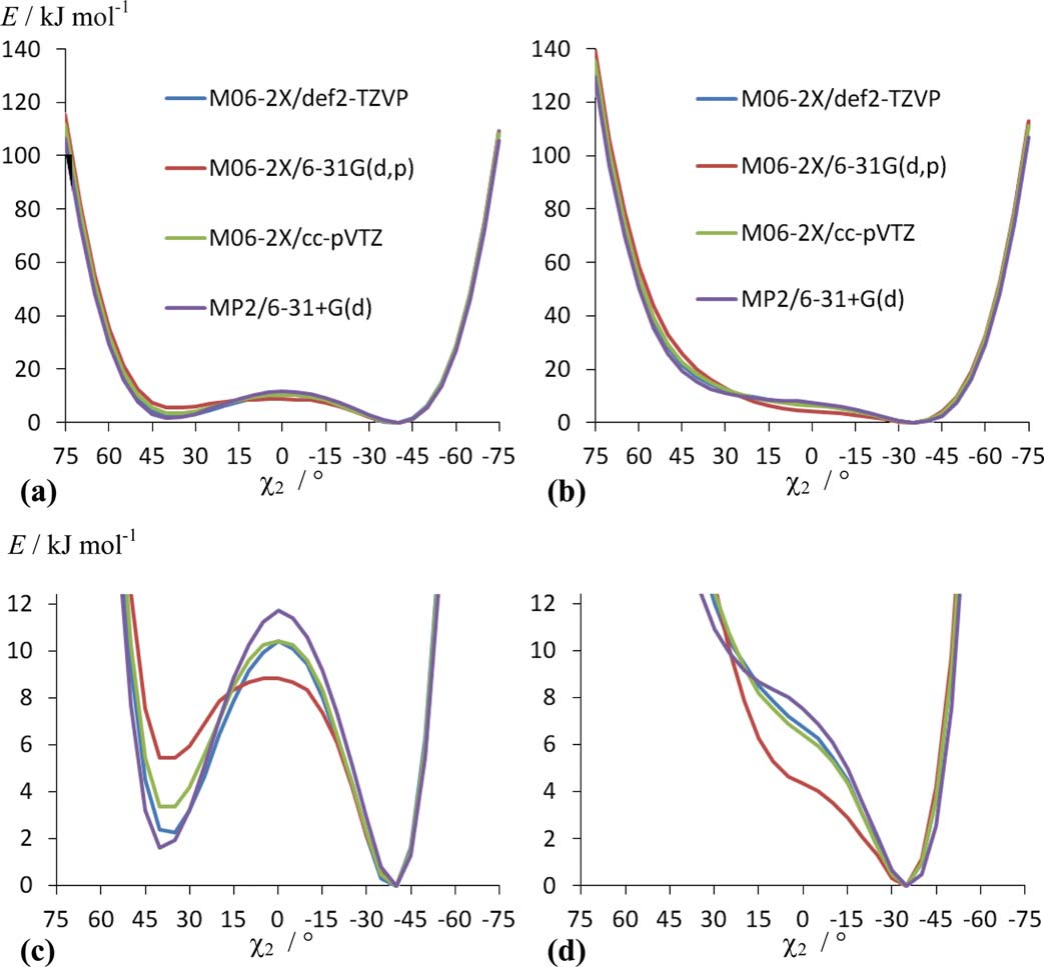
QM-predicted energy profiles as a function of the endocyclic pyrrolidine torsion angle χ_2_ in NAcPro (**a**) in water and (**b**) in the gas phase. Expansions of (a) and (b) are shown in (**c**) and (**d**), respectively.

After initial simplexed MD simulations, we considered QM optimizations of force field parameters followed by iterative MD simulations for further refinement of the force field parameters obtained from QM fittings. Four sets of QM calculations were considered to estimate the dependence of the results on the choice of the basis set and the QM method, as well as to assess the level of uncertainty involved: M06-2X/def2-TZVP, M06-2X/6-31G(d,p), M06-2X/cc-pVTZ and MP2/6-31+G(d). Based on previous studies,^[35,70]^ these QM methods and basis sets reproduce relative conformational energies and geometries in good agreement with experimental data. Calculations of 31 conformers of NAcPro were carried out in which the C^α^-C^β^-C^γ^- C^δ^ dihedral angle (χ_2_) was varied in 5° steps between −75° and +75°. The QM predicted energy profiles in the gas phase and in water (using IEFPCM)^63^,^64^ as a function of *χ*_2_ are compared in Figure 2. Considering relative energies of C^γ^-endo and C^γ^-exo conformers (with the corresponding *χ*_2_ values at ∼−40° and +40°, respectively), the experimentally measured ratio of two conformers in water (*x*_endo_=61% and *x*_exo_=39%)^34^ are best reproduced by IEFPCM(H_2_O) MP2/6-31+G(d) and M06-2X/def2-TZVP calculations [Fig. 2(c)]. The predicted populations of the C^γ^-endo were 66 and 71%, respectively, by IEFPCM(H_2_O) MP2/6-31+G(d) and M06-2X/def2-TZVP calculations. Thus, the results from these two sets of calculations were used in our further analysis.

The following merit function of Lindorff-Larsen *et al*. was used in our fittings^16^: (6)
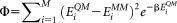
where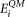 and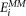 are the QM and molecular mechanics (MM) energies, respectively, and *M* is the number of conformations optimized at the QM level (31 in this case). The inverse temperature, β, is set to 1.0 mol kcal^−1^ (see discussion in Ref.^16^ regarding the choice of β value). Adopting the approach developed by Lindorff–Larsen *et al*.,^16^ the *E*^MM^ energy is given by the AMBER99SB energy, *E*^A99SB^, plus a new torsion term, that replaces the existing AMBER99SB torsion, *V*^A99SB^(θ): (7)
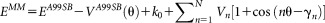
where *k*_0_ is a constant, the *V_n_*s are force constants in the cosine expansion including *N* terms and the γ_*n*_s are corresponding phases of the dihedral angle θ.

Simulated annealing fittings were employed to minimize Φ as a function of θ = χ_2_ with *N* = 3 by varying *V*_n_ and *γ*_n_ values in the torsional force-field term. In line with the approach used to modify the AMBER99SB backbone potential,^14^ we have assumed that *V*_n_ ≥ 0 kJ mol^−1^ and γ_n_ is either 0° or 180°. However, on fitting the gas phase data the predicted values of *V*_1_, *V*_2_ and *V*_3_ were 0 kJ mol^−1^ for both the MP2/6-31+G(d) and M06-2X/def2-TZVP data. We therefore consider only the IEFPCM(H_2_O) data below and any further reference to MP2/6-31+G(d) and M06-2X/def2-TZVP calculations assumes the use of the IEFPCM(H_2_O) method.

The values of the merit function Φ for the original AMBER99SB force field compared to the MP2/6-31+G(d) and M06-2X/def2-TZVP profiles were 2.84 and 2.24 kcal mol^−1^ (after *k*_0_-corrections according to Eq. (7)). On using simulated annealing fittings, these reduce to 2.39 kcal mol^−1^ for the parameter set (6) obtained from fittings to the MP2/6-31+G(d) profile and 1.93 kcal mol^−1^ for the parameter set (7) obtained from fittings to the M06-2X/def2-TZVP profile (Table1 and Fig. 2). In both cases, *V*_1_ = *V*_2_ = 0 and *V*_3_ ≠ 0 kJ mol^−1^. Such a result with only *V*_3_ ≠ 0 kJ mol^−1^ is not surprising, considering that χ_2_ in the pyrrolidine ring varies between ∼−40° and +40°. Only the *V*_3_ term (with γ_3_ = 0°) will have a maximum equal to *V*_3_ kJ mol^−1^ at χ_2_ = 0°, while the *V*_1_ and *V*_2_ terms (with γ_1_ = γ_2_ =180°) will show minima equal to 0 kJ mol^−1^ at χ_2_ = 0°. The value of *V*_3_ increases significantly compared to the original force field, which is in qualitative agreement with earlier results from simplexed MD simulations (parameter sets (1)–(5)) indicating to better agreement with experiment on increasing *V*_3_. From 200 ns MD simulations of NAcPro (Table1), the parameter set (7) from M06-2X calculations shows significantly better agreement with experiment than (6) derived from MP2 calculations.

Using the QM-derived parameter set (7) as a starting point, simplexed MD simulations were carriedout to optimize the value of *V*_3._ Initially, 40 ns long MD simulations were used at each step of simplexed MD simulations for merit function calculations. Parameter sets (8)-(23) were selected from these fittings with lowest merit function values for further 200 ns long MD simulations (Table1). On increasing the length of MD runs from 40 ns to 200 ns, the rms_*J*p_ values increase from 0.65–0.85 Hz to 0.73–1.08 Hz for parameter sets (8)-(23). The MD-averaged χ_m_ values predicted by these parameter sets (Table1) show better agreement with the experimental NMR value compared to the original AMBER99SB force field. However, it is likely that at relatively high values of *V*_3_ the short MD simulations used in simplexed MD fittings were not converged sufficiently. We therefore retain all new parameter sets (6)-(23) in our further analysis, as these provide sufficiently fine distribution of *V*_3_ values between 1.9 and 9.3 kJ mol^−1^. In addition, parameter sets (1)–(5) were also included in our further analysis.

### Single trajectory reweighting approach

The first application of the method relying on the energy-based reweighting approach^71^–^74^ to fittings of ^3^*J*-couplings of NAcPro with optimizations of three parameters *V*_1_, *V*_2_, and *V*_3_ led to unusually large values of *V*_2_ and *V*_3_ on using a 500-ns long MD trajectory with frames recorded every 0.04 ps: *V*_1_ = 0.0419, *V*_2_ = 22.3835, and *V*_3_ = 22.5864 kJ mol^−1^ with the rms of the fitting 0.79 Hz. As the predicted value of *V*_3_ is very high, significantly smaller number of the pyrrolidine ring transitions are expected in MD simulations compared to, for example, the number of peptide backbone transitions, which does not agree with experiment.^68^ As discussed by Li and Brüschweiler,^73^ the effectiveness of the reweighting scheme critically depends on the degree of overlap between the parent and the reweighted trajectories, since the reweighted procedure does not create any new conformations. On introducing a collectivity parameter κ with the requirement κ > 50% (see Eq. (2) and the discussion following it in Ref.^73^), a physically plausible solution was obtained from the 500 ns long parent trajectory of NAcPro in water: *V*_1_ = 0, *V*_2_ = 0.0009, and *V*_3_ = 2.3891 kJ mol^−1^. This set of parameters is essentially the same as (14) (Table1) and therefore is not included into our further analysis. On increasing the number of terms from three to six in Eq. (5), an alternative set of parameters was derived using the reweighting approach, which is included in Table1 as (24). Based on 200 ns MD simulations of NAcPro, this set of parameters performs slightly better than AMBER99SB and is therefore included into our further analysis.

### MD simulations of Gly-Pro-Gly-Gly

**Figure 3:**
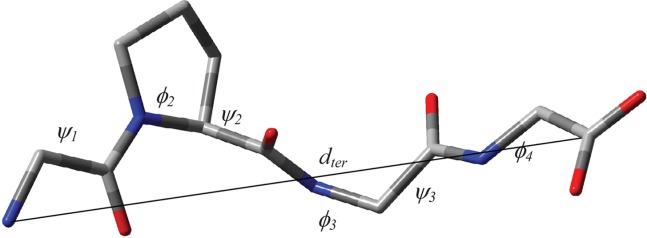
Unfolded conformation of Gly-Pro-Gly-Gly. Definitions of the backbone torsional angles and the distance between the terminal N and C atoms (*d*_ter_) are also shown. [Color figure can be viewed in the online issue, which is available at http://wileyonlinelibrary.com.]

For further examination, we carried out MD simulations of GPGG (Fig. 3) using force field parameters (1)-(24) and the original AMBER99SB force field. Note that AMBER99SB* or AMBER99SB-ILDN simulations would be the same in this case as the AMBER99SB simulation, as there are only Gly and Pro residues in GPGG. The recent study verifying different force fields using GPGG used 2 μs long MD simulations, which were sufficient for the majority of the force fields considered.^31^ However, on examination of the convergence of the population of the folded form against the length of the MD run for the AMBER99SB force field (Fig. 6 in Ref.^31^), it is clear that no significant change occurs in the population of the folded form after 600 ns. Thus, we carried out 800 ns long MD simulations for our analysis.

From the results obtained for the Pro ring in GPGG (Table2), all new parameter sets show better agreement with the experimental data with rms_*Jp*_ in the range between 0.48 and 0.62 Hz compared to the original force field (0.66 Hz). More importantly, all the tested sets provide higher values of χ_m_, (36.4°–41.3°) compared to the original set of parameters (35.3°). These results confirm that new parameter sets predict pyrrolidine ring geometries in better agreement with NMR, XRD and QM data compared to the original force field.

We have also analyzed NMR parameters dependent on the backbone conformation of GPGG. In particular, based on the analysis of NOE data for GPGG internuclear distances for seven proton pairs were measured previously.^36^ Averaged values of internuclear distances from MD runs were estimated over 800 ns time length for each of three MD simulations. The rms deviation between experiment and the MD predictions of distances (rms_*d*_) were calculated (Table SI, Supporting Information). In addition, four ^3^*J*_CH_ and two ^3^*J*_HH_ were available from NMR measurements for the GPGG backbone,^31^,^36^ which were used for NMR versus MD comparisons. As described previously,^31^ two empirical (corresponding to rms_*Je1*_ and rms_*Je2*_ in Table SI)^50^,^51^ and two QM-derived equations (corresponding to rms_*Jq1*_ and rms_*Jq2*_ in Table SI)^31^ were used to exclude possible model dependent deficiencies. For rms_*Jq1*_ and rms_*Jq2*_, we used B972 and B3LYP-predicted Karplus relationships,^31^ which have been shown to be sufficiently accurate.^75^–^81^ The results summarized in Table SI confirm that modifications of the Pro χ_2_ dihedral parameters do not cause any significant changes in the backbone conformations as there is a very good agreement for all the MD simulations when considering parameters averaged over backbone conformations. Similarly, the population of the U-shaped folded conformation of GPGG^36^ (*p*_f_, %) and the mean terminal N…C′ distance (*d*_ter_, Å) predicted by new parameter sets are in agreement with those predicted by the original AMBER99SB force field (Table3).

**Table 3:** Conformational Properties of GPGG Derived from MD Simulations^a^

	*p*_f_ (%)	*d*_ter_ (Å)	*N*^ψ1^	*N*^ψ2^	*N*^ϕ3^	*N*^ψ3^	*N*^χ1^	*N*^χ2^
AMBER99SB	17.5	8.5	18.59	5.36	17.97	25.36	51.35	81.25
1	17.2	8.5	18.84	5.17	18.04	25.10	45.51	65.79
2	16.4	8.5	18.91	5.32	18.52	24.71	43.31	59.44
3	17.0	8.5	18.46	5.48	18.13	24.59	40.73	54.20
4	19.1	8.3	18.82	5.29	17.23	26.10	44.93	62.69
5	18.7	8.4	18.16	5.62	17.83	25.39	33.52	41.27
6	13.6	8.8	19.61	5.30	19.14	23.09	1.01	1.02
7	14.6	8.7	19.33	5.36	18.72	23.50	4.62	4.70
8	16.1	8.5	18.54	5.69	18.68	25.13	30.89	37.23
9	16.6	8.5	18.76	5.41	18.26	24.51	7.94	8.20
10	17.0	8.5	19.07	5.44	18.34	24.69	14.98	16.15
11	17.0	8.5	18.83	5.64	18.43	24.70	21.95	24.50
12	17.5	8.4	18.73	5.41	17.88	25.37	19.20	21.15
13	16.0	8.6	19.42	5.13	18.35	23.68	18.03	19.65
14	16.1	8.6	18.97	5.31	18.51	24.35	30.14	35.76
15	17.0	8.4	19.14	5.74	18.52	24.64	27.14	31.65
16	16.1	8.6	18.67	5.42	18.47	24.36	24.14	27.38
17	16.3	8.6	18.95	5.49	18.42	24.16	5.30	5.43
18	16.0	8.6	18.94	5.49	18.58	24.33	3.46	3.50
19	15.6	8.6	18.83	5.26	19.03	24.25	3.88	3.92
20	14.6	8.7	19.39	5.46	19.39	23.95	13.40	14.21
21	16.3	8.6	19.50	5.52	18.50	24.21	11.06	11.68
22	15.5	8.6	19.08	5.28	18.93	24.17	11.97	12.72
23	15.6	8.6	18.79	5.51	18.44	24.06	15.71	16.95
24	16.8	8.5	18.88	5.22	18.22	24.58	0.73	0.74
25	17.4	8.5	18.99	5.27	17.91	24.84	14.18	15.21

a Shown are the population of the folded form (*p*_f_); the mean terminal N…C′ distance (*d*_ter_), the number of ψ_2_, ϕ_3_, ψ_3_ and χ_2_ torsional transitions per ns (*N*^ψ2^, *N*^ϕ3^, *N*^ψ3^ and *N*^χ2^, respectively). Frames recorded every 1 ps were used in the calculations of *N*^ψ2^, *N*^ϕ3^, *N*^ψ3^ and *N*^χ2^.

### Matching relative motional timescales from MD simulations and experiment

**Figure 4:**
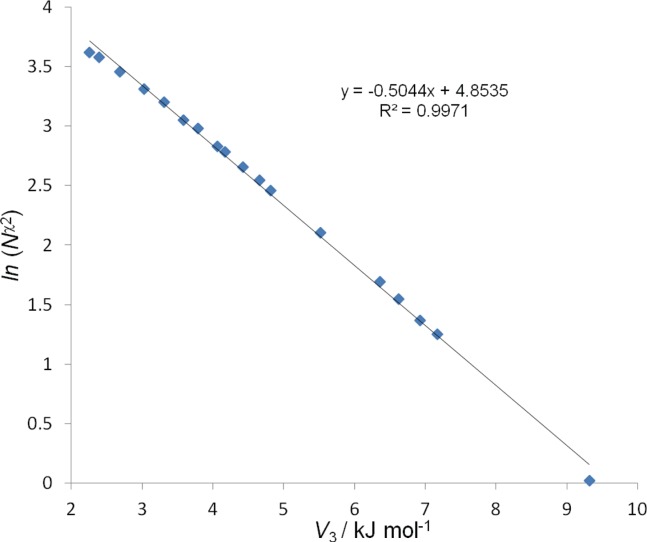
Plot of *ln* (*N*^χ2^) vs. *V*_3_ (in kJ mol^−1^) showing a linear dependence with *ln* (*N*^χ2^) = −0.5044 *V*_3_ + 4.8535 (*r*^2^ = 0.9971). [Color figure can be viewed in the online issue, which is available at http://wileyonlinelibrary.com.]

To identify which of the new parameter sets is likely to reproduce both structural and dynamics properties of Pro residues more accurately, we have considered timescales of motions in GPGG. First, we consider the number of the ψ_2_, ϕ_3_, ψ_3_ (see definitions of angles in Fig. 3), χ_1_ and χ_2_ (see definitions of angles in Fig. 1) torsional transitions per nanosecond (*N*^ψ2^, *N*^ϕ3^, *N*^ψ3^, *N*^χ1^, and *N*^χ2^ in Table3). As expected, the backbone transition numbers (*N*^ψ2^, *N*^ϕ3^ and *N*^ψ3^) are not affected by the change of the Pro torsional parameters, whereas moderate (*N*^χ2^ ≈ 41–66) and significant (*N*^χ2^ ≈ 1–37) decrease in the *N*^χ2^ values are observed for parameter sets (1)-(5) and (6)-(24), respectively, compared to the original force field (*N*^χ2^ ≈ 81). For new force field parameter sets containing only a single *V*_3_ term there is a linear relationship *ln* (*N*^χ2^) versus *V*_3_ (Fig. 4), as well as *ln* (*N*^χ2^) versus χ_m_ (Fig. S3, Supporting Information) and χ_m_ versus *V*_3_ (Fig. S4, Supporting Information). Thus, we can adjust the Pro sidechain torsional force field such that the timescale of the sidechain dynamics matches that from experiment.

Using ^13^C spin-lattice relaxation times measured for GPGG in water at 303 K,^68^ Mikhailov *et al*. have estimated that the auto-correlation time of the C^γ^—H bonds of Pro-2 is 27 ± 1.5 ps. As the accuracy of this type of measurements is critically dependent on the signal-to-noise ratio, we have repeated ^13^C spin-lattice relaxation time measurements of GPGG using a higher-field NMR spectrometer (14.1 T, 600 MHz ^1^H frequency) and a cryoprobe (Tables SII and SIII, Supporting Information). For the analysis of the *T*_1_ values and deriving correlation times, we have used the approach developed by Ernst *et al*.,^82^,^83^ which is different to that used by Mikhailov *et al*.^68^ The following equations were used to derive the correlation times for the overall (τ_c_) and the intramolecular ring interconversion (τ_e_) processes from the measured *T*_1_ relaxation times^82^–^84^: (8)
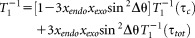

(9)
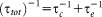

(10)
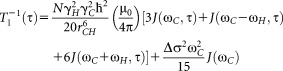

(11)
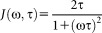
where Δθ is the jump angle of the C—H bond on conformational transition, γ_*H*_ and γ_*C*_ are gyromagnetic ratios of ^1^H and ^13^C, *ħ* is Planck's constant divided by 2π, *r_CH_* = 1.09 Å is the C—H bond length, Δσ is the chemical anisotropy of the ^13^C nucleus considered (see Experimental), *N* is the number of H atoms attached to the C atom. Note that in Eq. (8)), the sum of populations *x*_endo_ and *x*_exo_ is 1 (not in %).

The correlation time τ_c_ can be determined using the *NT*_1_ value (where *N* is the number of H atoms bonded to C) of the backbone C^α^ carbons, which are least affected by the intramolecular motions, hence better describe the overall motion of the molecule.^82^–^86^ In GPGG, *NT*_1_ values of C^α^ carbons are 1.146 s (Gly-1), 0.995 s (Pro-2), 1.106 s (Gly-3), and 1.836 s (Gly-4) (Table SIII, Supporting Information). The end residue backbone C^α^ carbons of Gly-1 and Gly-4 show the largest values, which suggest additional intramolecular dynamics for this carbon compared to mid-chain C^α^ carbons of Pro-2 and Gly-3. The minimum value of *NT*_1_ is observed for the C^α^ carbon of Pro-2, therefore we have used *T*_1_ of this backbone carbon to determine the correlation time τ_c_ for the overall motion. The likely intramolecular motion that can influence the *T*_1_ value for this carbon is the pyrrolidine ring interconversion. However, as estimated previously the jump angle Δθ is <5° for the C^α^ carbon of the pyrrolidine ring (see Table IX in Ref.^82^). Using Eqs. (8)– (11), it can be estimated that Δθ = 5° leads to only ∼0.4% increase in the *T*_1_ value and therefore can be neglected. From the *T*_1_ value of 995 ± 6 ms for the C^α^ carbon of Pro-2 in GPGG measured at 298 K for the 57 mM solution in D_2_O, the correlation time τ_c_ is 48.2 ± 0.3 ps. This value was used in the analysis of the *T*_1_ value for the C^γ^ carbon of Pro-2 in GPGG to determine the correlation time τ_e_ for the intramolecular ring interconversion (see below).

**Figure 5:**
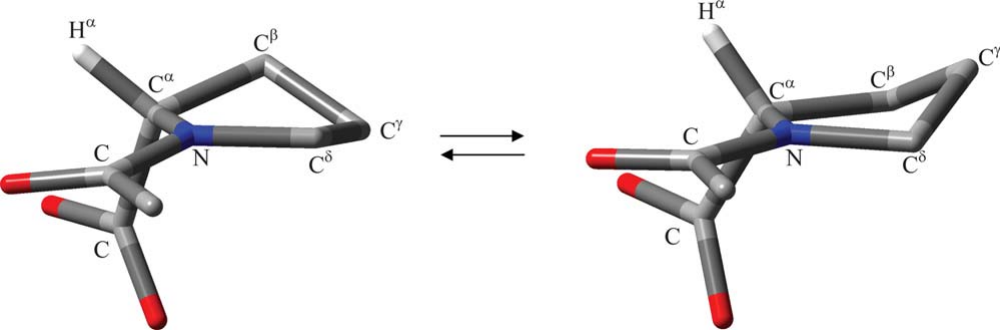
Interconversion between the C^γ^-endo (left) and C^γ^-exo (right) conformers of the Pro ring. For clarity of presentation, only one hydrogen atom, H^α^, is shown explicitly. [Color figure can be viewed in the online issue, which is available at http://wileyonlinelibrary.com.]

In Eq. (8), two terms are weighted by factors dependent on the populations of C^γ^-endo and C^γ^-exo ring conformers (*x*_endo_ and *x*_exo_, with *x*_endo_ + *x*_exo_ =1) and on the jump angle Δθ for a given C—H bond direction on changing the ring conformation. The largest jump angles are expected for C^γ^ carbon of the pyrrolidine ring. Thus, the *T*_1_ relaxation times of C^γ^ carbons (Tables SII and SIII, Supporting Information) were used for τ_e_ determinations. Madi *et al*. determined Δθ values using dihedral angles, which they estimated using the Karplus relationship.^82^ Because the accuracy of the Karplus relationship for predicting dihedral angles is relatively poor, we have taken a different approach, in which QM predicted geometries are used. Such an approach is supported by the finding that in the absence of relatively strong intermolecular interactions QM geometries reproduce accurately experimental molecular geometries derived from X-ray and neutron diffraction measurements.^70^ We used the two lowest energy conformations of NAcPro from M06-2X/def2-TZVP IEFPCM(H_2_O) calculations described above, the geometries of which were optimized without any restrictions. Additional frequency calculations were carried out to verify that the final structures correspond to true minima. The obtained structures correspond to C^γ^-endo- and C^γ^-exo-conformations of the pyrrolidine ring with *P*/χ_m_ values of 171.5°/39.3° and 16.5°/39.0°, respectively. As discussed previously,^82^,^83^ the most rigid part of the Pro ring in peptides is the C-N-C^α^-C fragment, where Cs are carbonyl carbons of COMe and COO in the case of NAcPro (see Fig. 5). We therefore overlaid the C^γ^-endo and C^γ^-exo conformations such that the rms deviations in the positions of four atoms of the C-N-C^α^-C fragment are minimal (Fig. S5, Supporting Information).^65^ The angle Δθ was then estimated as the angle between the corresponding C^γ^-H bond directions in two conformations. The values of Δθ determined for the C^γ^-H^γ2^ and C^γ^-H^γ3^ bonds were 82.65° and 82.47° with the average value of 82.56°, which was used as a fixed value of Δθ in our fittings using *T*_1_ relaxation times of C^γ^ carbons. The populations of C^γ^-endo and C^γ^-exo ring conformers are known from the analysis of ^3^*J*_HH_ coupling constants measured at 298 K (Table2)^35^ and are assumed to be temperature independent. With these restrictions in place, the correlation time τ_e_ for the intramolecular ring interconversion process were determined using the measured *T*_1_ values for C^γ^ carbons at different temperatures. From the comparison of the above Eq. (8) and Eq. (37) of Lipari and Szabo,^59^ the generalized order parameter is dependent on the populations of conformers and the jump angle Δθ in the case of the two-site jump model and can be calculated using the following relationship: (12)
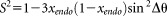


For *x*_endo_=0.543 and Δθ=82.56°, the calculated experimental value of *S*^2^ is 0.27.

Using the measured *T*_1_ values for C^α^ and C^γ^ carbons of Pro in GPGG for the 214 m*M* solution of D_2_O (Table SII, Supporting Information) the values of correlation times *τ*_c_ and *τ*_e_ were determined at different temperatures (Table SIV, Supporting Information). Assuming Arrhenius dependence of correlation times [*τ* = *τ*^0^ *exp* (*E*_a_/*RT*)], activation parameters are *E*_a_ = 16.4 ± 1.2 kJ mol^−1^ and *τ*_e_^0^ = (4.1 ± 1.6) × 10^−14^ s for the pyrrolidine ring interconversions. To estimate errors in activation parameters, we have excluded two highest and two lowest temperatures from consideration which led to *E*_a_ variations between 15.6 and 17.6 kJ mol^−1^ and *τ*_e_^0^ variations between 2.5 × 10^−14^ to 5.5 × 10^−14^ s. The estimated correlation time τ_e_ for the C^γ^—H bond movements in Pro-2 of GPGG as a result of the pyrrolidine ring interconversion is 27.2 ps at 303 K, which is in good agreement with the value of 27 ps reported by Mikhailov *et al*.^68^

Our MD simulations were carried out at 298 K. Using the *T*_1_ value of 898 ± 4 ms for the C^γ^ carbon of Pro-2 in GPGG measured at 298 K for the diluted 57 m*M* solution of GPGG in D_2_O, we have estimated the correlation time *τ*_e_ for the C^γ^—H bond reorientations in Pro-2 of GPGG as a result of the pyrrolidine ring interconversion as 29.7 ± 0.4 ps, which is slightly smaller than the value of *τ*_e_ calculated as 30.3 ps using the activation parameters reported above for the 214 m*M* solution. As higher concentrations may in principle lead to partial self-associations of peptides,^87^ we have used the experimental value of *τ*_e_ = 29.7 ps at 298 K as a reference point for our MD simulations. From the analysis of *τ*_e_ calculated for 14 parameter sets with a single non-zero *V_3_* term (*γ*_3_ = 0°, Table4), there is a linear correlation (Fig. S6, Supporting Information): *V_3_* (in kJ mol^−1^) = 1.9272 *ln τ*_e_ (in ps) – 2.1881 (with *r*^2^ = 0.9975). Using this relationship, we estimate *V_3_* = 4.3474 kJ mol^−1^ for *τ*_e_ = 29.7 ps. For backward verification, the 800-ns long MD simulation at 298 K with *V_3_* = 4.3474 kJ mol^−1^ (γ_3_ = 0°) predict τ_e_ = 28.7 ps and *S*^2^ = 0.29, in close agreement with the experimentally measured values of τ_e_ = 29.7 ps and *S*^2^=0.27. This parameter set (denoted as (25) in Tables5) is selected as the final solution which reproduces the experimental structural (Tables2 and 3) and dynamic properties (Tables3 and 4, Fig. S7) of the sidechain of the Pro residue significantly better than the original AMBER99SB force field.

**Table 4:** Intramolecular Autocorrelation Times τ_*e*_ (in ps) and Order Parameters *S*^2^ for the C^γ^—H Bond Reorientations of Pro in GPGG as Predicted by 800-ns MD simulations

Parameter set	*V*_3_, (kJ mol^−1^)	*S*^2^	τ_*e*_, ps	rms^a^
AMBER99SB	0.75312	0.33	4.1	0.0017
1	1.37935	0.32	5.6	0.0020
4	1.06169	0.32	5.9	0.0020
2	1.29055	0.31	6.4	0.0022
3	1.75728	0.31	7.1	0.0023
5	1.95811	0.69	11.0	0.0011
8	2.25938	0.30	11.1	0.0029
14	2.3954	0.30	11.6	0.0037
15	2.6885	0.30	12.9	0.0029
16	3.028	0.29	15.2	0.0033
11	3.30976	0.29	16.9	0.0034
12	3.58557	0.29	20.0	0.0040
13	3.79243	0.28	21.6	0.0040
23	4.06	0.28	25.4	0.0043
10	4.17167	0.29	26.2	0.0040
20	4.42712	0.28	29.3	0.0046
22	4.6633	0.28	34.0	0.0049
21	4.816241	0.28	36.6	0.0048
9	5.51626	0.27	54.3	0.0059
17	6.35714	0.27	82.8	0.0076
7	6.61951	0.28	91.7	0.0079
19	6.92437	0.27	112.0	0.0085
18	7.17114	0.27	124.2	0.0091
6	9.31503	0.25	440.2	0.0147
24	7.4146^b^	0.30	531.5	0.0177
25	4.3474	0.29	28.7	0.0045
NMR	—	0.27(1)	29.7(4)	—

a The fitting errors (rms, arbitrary units with *C*(*t*) = 1 at *t* = 0 ps) are shown.

b The sum of *V*_4_, *V*_5_ and *V*_6_ is shown.

### Force field phase variations

In another set of optimizations we considered variations of both the *V*_3_ force constant and the phase γ_3_. The value of *V*_3_ was varied between 1 and 5 kJ mol^−1^ with a step of 1 kJ mol^−1^, while the value of γ_3_ was varied between −50 and 50° with a step of 10°. The results of 700 ns long MD simulations for each pair of *V*_3_ and *γ*_3_ values are summarized in Tables SV–SVIII in Supporting Information. Over four parameters considered (rms_*J*p_, *x*_endo_, *τ*_e_ and *S*^2^), the force field with *V*_3_ = 4.0 kJ mol^−1^ and *γ*_3_ = 0° shows the best agreement with experiment. This additional grid search analysis allowed us to confirm that the above optimization leading to *V*_3_ = 4.3474 kJ mol^−1^ and *γ*_3_ = 0° is the unique solution in the two-dimensional (*V*_3_,γ_3_)-parameter space.

### Influence on the backbone conformation

To examine the influence of the new sidechain parameter set on the protein backbone conformations and dynamics, we have carried out 1-μs long MD simulations of ubiquitin. Three Pro residues of ubiquitin—Pro-19, Pro-37, and Pro-38—were considered, conformational characteristics of which are compared in Table SIX (Supporting Information). Compared to the original force field, the parameter set (25) lead to higher *χ*_m_ values (38.8–39.5°), which are in better agreement with experimental XRD data.^48^,^88^ In particular, the solid-state values of *χ*_m_ are 42.5° (Pro-19), 44.2° (Pro-37), and 45.2° (Pro-38).^88^

Unlike Pro-19 and Pro-37, the pyrrolidine ring of Pro-38 in ubiquitin is in predominantly C^γ^-exo conformation according to MD simulations (Table SIX), which is in agreement with the finding that in Xaa-Yaa-Gly triplets of collagen the Pro ring prefers the endo pucker (i.e., C^γ^-endo conformation) in the X position, while in the Y position it prefers the exo pucker.^89^,^90^ In principle, this can be verified experimentally by measuring accurate values of ^3^*J*_HH_-couplings of the pyrrolidine rings in ubiquitin. However, pyrrolidine cyclic protons usually show strongly-coupled ^1^H NMR spectra due to small chemical shift differences for methylene protons in β and γ positions.^34^ Accurate measurements of *J*_HH_-couplings would therefore require a full lineshape analysis, which is complicated by strongly overlapping spectra in the case of proteins.

The values of *N*^χ2^ in ubiquitin prolines are in good agreement with those predicted for the Pro residue in GPGG, although the number of *χ*_2_ transitions decreases significantly in Pro-38, which is likely caused by the Pro-37 residue preceding Pro-38. We have compared three experimental ^3^*J*(C′,H^α^) couplings of 1.22 Hz (Pro-19), 1.71 Hz (Pro-37), and 1.06 Hz (Pro-38) in ubiquitin^37^,^38^ with those calculated from MD simulations of ubiquitin using Karplus parameters, derived empirically^50^ and from DFT B3LYP/EPR-III calculations.^31^ Compared to the AMBER99SB*-ILDN calculations, the parameter set (25) lead to only small variations in ^3^*J* values (Table SIX, Supporting Information). This result confirms that the changes in the sidechain dynamics interchanging the C^γ^ atom position below and the above the C^α^-N-C^γ^ plane cause only small changes in the torsional angle H^α^-C^α^-N-C (Fig. 5 and Fig. S5).

Finally, the performance of parameter sets AMBER99SB*-ILDN and (25) were compared using experimental values of five different types of backbone ^3^*J*-couplings, each of which has been determined for 60–67 amino acid residues in ubiquitin.^37^,^38^ On calculating the MD-predicted averaged ^3^*J*-couplings we have considered up to four different sets of Karplus parameters for each type of ^3^*J* coupling.^49^,^50^ From the results summarized in Table SX (Supporting Information), both force fields reproduce ^3^*J* couplings equally well, confirming that the new Pro torsion potential does not cause undesirable side effects on the backbone conformations compared to the original force field, the performance of which has been verified extensively.^3^,^4^,^14^,^15^,^19^–^33^

### Force field validation

As an independent test, we have used NMR data and MD simulations of Val-Ala-Pro-Gly (VAPG). In Table5, we compare conformational populations and geometries of the Pro ring in VAPG in water as predicted by NMR and by 800-ns long MD simulations. The rms_*J*p_ values relative to experimental values of 10 ^3^*J*_HH_-couplings show that the new force field (25) reproduces better the experimentally measured values than the original force field. The value of *χ*_m_ serves as a measure of non-planarity of the five-membered ring. The results confirm that the new force field (25) leads to significantly improved agreement with experiment compared to the original force field AMBER99SB.

**Table 5:** Conformational Populations and Geometries of the Pro ring in Aqueous Solutions of Peptides from NMR and MD Simulations Using Different Sets of Torsional Parameters for the Pro residue^a^

Peptide	Force field	*P*_exo_ (°)	*P*_endo_ (°)	*χ*_m_ (°)	*x*_endo_ (%)	rms_*J*p_ (Hz)	*S*^2^	*τ*_e_(ps)
Val-Ala-Pro-Gly	AMBER99SB	14	178	36.3	62.6	0.867	0.35	4.2
(VAPG)	25	13	180	38.8	62.6	0.801	0.31	28.6
	NMR^35^	14(4)	187(2)	41.0(4)	52.3(2)	0.47^b^	0.26(1)	30.7(5)
*cis*-VAPG	AMBER99SB	23	174	36.8	71.1	1.318	0.41	3.3
	25	19	176	39.4	73.8	1.004	0.41	20.9
	NMR^35^	20(9)	177(8)	42(2)	82.6(9)	0.59^b^	0.58(3)	22(2)
Gly-Pro-Phe	AMBER99SB	15	179	35.9	61.2	0.864		
(GPF)	25	14	179	38.8	61.6	0.802		
	NMR	22(6)	183(2)	39.8(8)	68(1)	0.31^b^		
Angiotensin II	AMBER99SB	15	178	35.5	68.2	1.320	0.26	8.4
	25	12	180	38.8	65.0	1.033	0.23	33.1
	NMR	14(8)	198(6)	42(2)	53(1)	0.38^b^	0.26(1)^c^	32(4)^c^

a 1.5 μs MD simulations for angiotensin II and 800 ns for other peptides were analyzed.

b The rms deviation for NMR is for fittings of experimental ^3^*J*_HH_ values using Eqs. (8C) and (8D) of Haasnoot *et al*.^46^ on the assumption of a two-site conformational exchange between C^γ^-endo and C^γ^-exo conformers and χ_m_^endo^= χ_m_^exo^.^34^

c The values and uncertainties were determined using *T*_1_=386 ± 12 ms for ^13^C^γ^ of Pro-7. From M06-2X/def2-TZVP calculations of GPF, the jump angle Δθ was 83.16°.

In terms of motional dynamics, the predicted values of the correlation time and generalized order parameter for the Pro ring interconversion at 298 K are 4.2 ps and 0.35, respectively, according to the 800-ns MD simulations at 298 K using the original AMBER99SB force field. The predicted value of *τ*_e_ is significantly different from the value measured experimentally in this work using *T*_1_(^13^C) values at 298 K (Table SXI, Supporting Information): 30.7 ± 0.5 ps for the 77 m*M* solution of VAPG in H_2_O:D_2_O (9:1). For *x*_endo_ = 0.523 and Δθ = 82.56°, the estimated experimental value of *S*^2^ is 0.26. Note that in VAPG, the *NT*_1_ values of C^α^ carbons are 0.751 s (Val-1), 0.614 s (Ala-2), 0.641 s (Pro-3) and 1.142 s (Gly-4) (Table SXI, Supporting Information). Judging by *NT*_1_ values, the C^α^ site of Ala is least affected by intramolecular motions, thus the *T*_1_ value of this carbon was used to determine the correlation time for the overall molecular motion (*τ*_c_ = 82.8 ± 0.7 ps). The corresponding values predicted by the new force field are *τ*_e_ = 28.6 ps and *S*^2^=0.31, which are in good agreement with experiment.

Although we have primarily focused on force field optimizations for the *trans*-rotamer about the bond preceding the Pro residue, it would be interesting to verify whether the new force field would offer any improvements for the *cis*-rotamer compared to the original force field. In the case of *cis*-VAPG (with the *cis*-orientation of the CH_2_ group of Gly and the CO group of Pro), the MD-predicted ^3^*J*_HH_ couplings by the new force field (25) show improved agreement with experimental values of ^3^*J*_HH_ couplings compared to the original force field as judged by the rms_*J*p_ values: 1.00 Hz and 1.32 Hz for force fields (25) and AMBER99SB. However, the agreement with the experiment is not as good as for the *trans*-VAPG considered above due to the lower value of the predicted population of the C^γ^-endo conformer by the new force field (74%, as opposed to the experimental value of 83%). The difference in the predicted population by the new force field is further amplified in the predicted value of *S*^2^=0.41 (experimental value 0.58), as *S*^2^ is proportional to the product of *x*_endo_ and (1 - *x*_endo_). At the same time, the predicted value of τ_e_ = 20.9 ps by the new force field is in good agreement with the experimental value of 22 ± 2 ps. For comparison, the predicted values of *S*^2^ and τ_e_ by AMBER99SB are 0.41 and 3.3 ps, respectively.

The change of the amino acid residue proceeding the Pro residue to Phe has been shown to lead to the increased population of the C^γ^-endo conformer.^91^ We have re-determined conformational characteristics of the Pro residue in Gly-Pro-Phe (GPF) using experimental values of all ten ^3^*J*_HH_ couplings reported by Anteunis *et al*.^91^ and the least squares fitting procedure described previously.^34^ The results summarized in Table5 confirm that the content of the C^γ^-endo conformer increases in GPF (*x*_endo_ = 68.0%) compared to that in GPGG and VAPG. However, the degree of change is not as significant as previously predicted (*x*_endo_ = 85%) using Karplus relations of Pogliani *et al*.^92^ In Table5, we compare conformational populations and geometries of the Pro ring in GPF in water from 800 ns long MD simulations and experiment. As in the case of tetrapeptide VAPG above, the rms_*J*p_ values relative to experimental values of ten ^3^*J*_HH_-couplings show that the new force field (25) reproduces better the experimentally measured values than the original force field. The higher values of *x*_endo_ and *χ*_m_ compared to the original force field are also in better agreement with experiment (Table5).

We have also analyzed NMR data and MD simulations of octapeptide angiotensin II (Asp-Arg-Val-Tyr-Ile-His-Pro-Phe, Fig. S8 in Supporting Information). After initial assignments of peaks in ^1^H and ^13^C spectra of 16 m*M* solution of angiotensin II in D_2_O using 2D NMR spectra (Tables SXII and SXIII, Supporting Information), full ^1^H NMR lineshape analysis was carried out to determine vicinal ^3^*J*_HH_ couplings of the Pro-7 sidechain (Fig. S9 and Table SXIV, Supporting Information), which were subsequently analyzed to estimate conformational characteristics of the pyrrolidine ring of Pro-7 in angiotensin II. In addition, ^13^C spin-lattice relaxation times were measured at 298 K (Table SXV in Supporting Information), which allowed to measure values of *S*^2^ and τ_e_. As in the case of GPGG and VAPG discussed above, the *T*_1_ values of the backbone C^α^ carbons show clear decrease towards the mid-chain residues (in ms next page):

Asp Arg Val Tyr Ile His Pro Phe
520 355 347 310 324 327 372 448

The minimum value of *T*_1_ observed for the C^α^ carbon of Tyr-4 suggests that this site is least affected by intramolecular motions. It is therefore best suited for determining the correlation time τ_c_ of the overall molecular motion. From Eqs. (8)– (11), the value of τ_c_ corresponding to *T*_1_ = 310 ± 3 ms is 246 ± 6 ps. This value was used in the analysis of the *T*_1_ value for the C^γ^ carbon of Pro-7 in angiotensin II to determine the correlation time τ_e_ for the intramolecular ring interconversion (see below).

To estimate the jump angle Δθ in angiotensin, we have used M06-2X/def2-TZVP calculations of GPF with the Phe residue following Pro as in angiotensin II. After overlaying the C^γ^-endo- and C^γ^-exo-conformations of GPF such that the rms deviations in the positions of four atoms of the C-N-C^α^-C fragment are minimal, the jump angle Δθ was determined as 83.16° (82.97° for C^γ^-H^γ2^ and 83.34° for C^γ^-H^γ3^), which was used as a fixed value of Δθ in our fittings *T*_1_ relaxation data.

In Table5, we compare conformational populations and geometries of the pyrrolidine ring of angiotensin II in water determined by NMR and by 1500-ns long MD simulations. The rms_*J*p_ values relative to experimental values of 10 ^3^*J*_HH_-couplings show that the new force field (25) with rms_*J*p_ = 1.03 Hz reproduces the experimentally measured values better than the original force field with rms_*J*p_ = 1.32 Hz. For the pseudorotation amplitude *χ*_m_, the results confirm that the new force field (25) leads to significantly improved agreement (*χ*_m_ = 38.8°) with experiment (*χ*_m_ = 42° ± 2°) compared to AMBER99SB (*χ*_m_ = 35.5°). Regarding motional dynamics (Table5), the timescale of motion is reproduced significantly better by the new force field (25). The corresponding values of τ_e_ are 8.4, 33.1 and 32 ± 4 ps for AMBER99SB, the new force field (25) and experiment, respectively.

Finally, the relative experimental values of overall and internal correlation times *τ*_c_/*τ*_e_ were 48.2 ps/29.7 ps in GPGG, 82.8 ps/30.7 ps in VAPG and 246 ps/32 ps in angiotensin II. These clearly show that despite the fivefold increase in the correlation time of the overall motion, the timescale of the internal motion remains essentially unchanged in these peptides of varying size. Thus, it is likely that the overall molecular motions and the intramolecular dynamics of the Pro ring are independent in the peptides considered.

### Force field parameters of hydroxyproline

**Figure 6:**
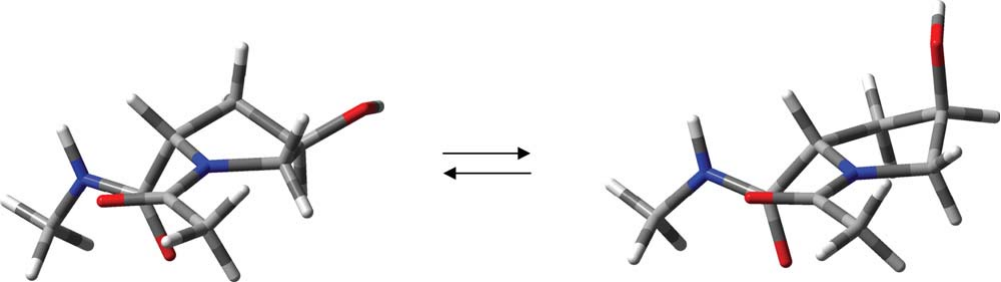
The C^γ^-endo (left) and C^γ^-exo (right) conformers of Ace-Hyp-NHMe (AHM). [Color figure can be viewed in the online issue, which is available at http://wileyonlinelibrary.com.]

Together with Pro and Gly, the 4-hydroxyl-L-proline residue (Hyp) is one of the main building blocks in collagen,^89^,^90^,^93^ although it is not included in the list of 20 natural amino acid residues. In the GROMACS implementation of AMBER99SB, the force field parameters of Mooney *et al*. is used for the N-C^δ^-C^γ^-O torsion of Hyp,^89^ although reparameterization by Park *et al*.^90^ has been shown to reproduce the experimentally observed preference of the C^γ^-exo conformer in Hyp over the C^γ^-endo conformer better than that of Mooney *et al*.^89^ Our MD simulations carried out for Ace-Hyp-NHMe (AHM, Fig. 6) are in agreement with these findings (Table6). The predicted population of the C^γ^-endo conformer is 51.4% on using parameters of Mooney *et al*., while the smaller value of 6.7% predicted by the Hyp parameters of Park *et al*. is in good agreement with the experimental value of 12%. Similarly, the experimental ^3^*J*_HH_ couplings of the Hyp ring are better reproduced by parameters of Park *et al*. (rms_*J*p_=1.05 Hz) compared to that of Mooney *et al*. (rms_*J*p_=2.72 Hz). However, the *χ*_m_ values by both parameter sets show flattened ring geometries compared to experiment (Table6). Furthermore, the predicted motional characteristics of the ring dynamics by both parameter sets are in sharp contrast with experiment, showing significantly higher frequencies of ring interconversions. In particular, the correlation times of the ring interconversions (τ_e_) are 7.8 ps (Mooney *et al*.), 1.5 ps (Park *et al*.) and 82.6 ps (experiment).

**Table 6:** Conformational Populations and Geometries of the Hyp Ring in AHM in Water from NMR and 1.5-μs Long MD Simulations Using Various Sets of Torsional Parameters for the Hyp Residue

Force field	*V*_3_ (kJ mol^−1^)	*P*_exo_ (°)	*P*_endo_ (°)	*χ*_m_ (°)	*x*_endo_ (%)	rms_Jp_ (Hz)
AMBER99SB^a^	0.65084	14	177	35.0	51.4	2.721
AMBER99SB^b^	_b_	14	167	34.6	6.7	1.046
h1	1.7	13	179	38.4	50.8	2.652
h2	2.7	14	181	38.9	48.6	2.528
h3	3.7	15	182	39.3	45.0	2.321
h4	4.7	15	183	39.8	44.6	2.296
h5	5.7	16	184	40.1	41.0	2.085
h6	6.7	16	185	40.6	38.6	1.946
h7	7.7	16	186	40.9	35.9	1.941
h8	8.7	17	186	41.2	33.4	1.641
h9	9.7	18	187	41.4	32.3	1.579
h10	10.7	18	188	41.6	36.6	1.828
h11	11.7	18	188	41.8	22.7	1.026
h12	12.7	18	189	42.2	27.6	1.302
NMR	—	12(1)	215(9)	42(2)	11.9(8)	0.344^c^

a Apart from the original AMBER99SB force fields using the Hyp force field parameters of Mooney *et al*.^89^ and Park *et al*.,^90^ all other models use *V*_3_=4.3474 kJ mol^−1^ (γ_3_ = 0°) for the endocyclic C—C—C—C (*χ*_2_) torsion of the Hyp residue of AHM.

b The modified Hyp force field parameters of Park *et al*. were used as a Ryckaert–Bellemans function with *C*_0_ = 0.6527 kJ mol^−1^ and *C*_2_ = 12.46832 kJ mol^−1^.^90^

c The rms deviation for NMR is for fittings of experimental ^3^*J*_HH_ values using Eqs. (8C) and (8D) of Haasnoot *et al*.^46^ assuming a two-site exchange between C^γ^-endo and C^γ^-exo conformers and *χ*_m_^endo^= *χ*_m_^exo^.^34^

We have optimized the force field parameters for the hydroxyproline N-C^δ^-C^γ^-O torsional angle (denoted as χ_h_) to better match the dynamics characteristics of the Hyp sidechain. The new force field (25) for the C—C—C—C (χ_2_) torsion was used as a fixed constant (*V*_3_ = 4.3474 kJ mol^−1^ and γ_3_ = 0°) in these optimizations for the Hyp residue. In the original AMBER99SB force field *V*_3_ = 0.65084 kJ mol^−1^ and γ_3_ = 0° for the hydroxyproline N-C^δ^-C^γ^-O (χ_h_) torsion. Initially, 1.5-μs MD simulations were considered in which the value of *V*_3_ for *χ*_h_ was gradually increased (Table6). This showed that the population *x*_endo_ approaches the experimental value at only very high values of *V*_3_ (see Table6), at which even 1.5 μs MD simulations may not be sufficient for the convergence of the predicted population.

Similar to the Pro residue considered above, we used QM calculations to fit the *χ*_h_ parameters in Hyp. The M06-2X/def2-TZVP IEFPCM(water) calculations of 26 conformers of AHM were carried out in which the N-C^δ^-C^γ^-O dihedral angle was varied in 5° steps between 52.8° and 177.8°. Simulated annealing fittings were employed to minimize the value of merit function Φ [Eq. (6)] as a function of θ = χ_h_ by varying *V*_3_ values (γ_3_ = 0°) and *k*_0_ [Eq. (7)]. This led to *V*_3_ = 5.5574 kJ mol^−1^ with only small improvement in the value of Φ (0.44 kcal mol^−1^) compared to the original force field with the Hyp parameters of Mooney *et al*. (0.46 kcal mol^−1^). The QM-optimized value is close to the value of *V*_3_ = 5.7 kJ mol^−1^ in Table6, which predicts very high value of *x*_endo_ compared to experiment. Therefore, no new MD simulations were carried out.

In a new set of optimizations we considered variations of both the *V*_3_ force constant and its phase γ_3_. The results of 600-ns long MD simulations for each pair of *V*_3_ and γ_3_ values are summarized in Tables SXVI–SXIX (Supporting Information). Over four parameters considered (rms_*J*p_, *x*_endo_, τ_e_ and *S*^2^), the force field with *V*_3_ = 5.3 kJ mol^−1^ and γ_3_ = 30° shows the best agreement with experiment. From the spin-lattice relaxation time measurements for a 59 m*M* solution of AHM in D_2_O at 298 K, *τ*_c_ = 32.8 ± 0.5 ps, *τ*_e_ = 82.6 ± 2.8 ps and *S*^2^ = 0.69 ± 0.01 (full NMR data for AHM is included in Tables SXX–SXXII in Supporting Information). The *τ*_e_ values for the force constants *V*_3_= 4.3, 5.3, and 6.3 kJ mol^−1^ at *γ*_3_ = 30° show a satisfactory linear relationship: *V_3_* (in kJ mol^−1^) = 3.6404 *ln* τ_e_ (in ps) −10.555 (with *r*^2^ = 0.9968). Using this relationship, we estimate *V_3_* = 5.5138 kJ mol^−1^ for the experimental value of τ_e_ = 82.6 ps. This value of *V*_3_ together with the phase γ_3_ = 30° was used for our further verifications (referred to as parameter set (h13)). A 1.5-μs long MD simulation using force field (h13) for χ_h_ of Hyp (with force field (25) for the χ_2_ potential) confirmed the improvement of the parameterization of the χ_h_ potential, as *S*^2^ is 0.69 and τ_e_ = 77.6 ps compared to the original AMBER99SB force field with *S*^2^ = 0.34 and τ_e_ = 7.8 ps and the experimental values of *S*^2^ = 0.69 and τ_e_ ≈ 83 ps (Table7). Also, the predicted *x*_endo_ population is 9.6%, which is in close agreement with the experimental value of 11.9%. In addition, the *χ*_m_ value increases from 35.0° for AMBER99SB to 39.5° for (h13), which compares better to the experimental estimate of 42° ± 2°. As expected, these improvements are reflected in the considerable reduction in the rms_*J*p_ value, which decreases from 2.72 Hz for AMBER99SB with the Hyp parameters of Mooney *et al*.^89^ to 0.62 Hz for model (h13).

**Table 7:** Conformational Populations and Geometries of the Hyp Ring in AHM and AHG in Water from NMR and 1500-ns MD Simulations Using Different Sets of Torsional Parameters for the Hyp Residue

Peptide	Force field	*P*_exo_ (°)	*P*_endo_ (°)	*χ*_m_ (°)	*x*_endo_ (%)	rms_Jp_ (Hz)	*S*^2^	τ_e_ (ps)
AHM	AMBER99SB^a^	14	177	35.0	51.4	2.721	0.34	7.8
	AMBER99SB^a^^b^	14	167	34.6	6.7	1.046	0.78	1.5
	h13	14	183	39.5	9.6	0.624	0.69	77.6
	NMR	12(1)	215(9)	42(2)	11.9(8)	(0.34)^c^	0.69(1)^d^	83(3)^d^
AHG	AMBER99SB^a^	14	176	35.0	51.2	2.597	0.34	8.8
	AMBER99SB^a^^b^	15	162	34.6	6.6	1.018	0.78	1.7
	h13	14	183	39.6	9.4	0.635	0.70	79.9
	NMR	12(1)	213(8)	42(2)	13.9(6)	(0.36)^c^	0.65(1)^d^	80(4)^d^

a Apart from the original AMBER99SB force fields using the Hyp force field parameters of Mooney *et al*.^89^ and Park *et al*.,^90^ all other models use *V*_3_=4.3474 kJ mol^−1^ (γ_3_ = 0°) for the endocyclic C—C—C—C (*χ*_2_) torsion of the Hyp residue.

b The modified Hyp force field parameters of Park *et al*. were used as a Ryckaert–Bellemans function with *C*_0_ = 0.6527 kJ mol^−1^ and *C*_2_ = 12.46832 kJ mol^−1^.^90^

c The rms deviation for NMR is for fittings of experimental ^3^*J*_HH_ values assuming a two-site exchange between C^γ^-endo and C^γ^-exo conformers and χ_m_^endo^= χ_m_^exo^.^34^

d The values and uncertainties were determined using *T*_1_ for ^13^C^γ^ of Hyp in 59 m*M* D_2_O solutions. From M06-2X/aug-cc-PVTZ calculations of AHM, the jump angle Δθ used for determining *S*^2^ and τ_e_ in AHM and AHG was 82.64°. The τ_c_ values determined using *T*_1_ for ^13^C^α^ of Hyp were 32.8 ± 0.5 ps for AHM and 43.5 ± 0.6 ps for AHG.

Further independent validation for the hydroxyproline parameters was carried out using 1.5-μs long MD simulations of *N*-acetyl-4-hydroxy-l-proline-glycine (Ace-Hyp-Gly, AHG, Fig. 6; full NMR data is included in Tables SXX–SXXII, Supporting Information). The new force field (h13) for the *χ*_h_ torsion together with the force field (25) for the *χ*_2_ endocyclic torsion shows a much improved agreement with experiment compared to the original force field AMBER99SB (Table7). The value of *χ*_m_ increases from 35° and 34.6° for the AMBER99SB force field with the Hyp parameters of Mooney *et al*.^89^ and Park *et al*.,^90^ respectively, to 39.6°. For comparison, *χ*_m_ = 42° ± 2° based on the analysis of the experimental NMR data. The predicted value of *x*_endo_ also shows improved agreement with experiment, that is, the experimental value of 13.8% ± 0.5% is reproduced as 9.4% by the new force field. This is also reflected in the reduced rms_*J*p_ value which is 0.64 Hz (Table7). By far the largest improvement is obtained for dynamics characteristics of the hydroxyproline ring interconversion. For example, the original force field using the Hyp parameters of Mooney *et al*. predicts *τ*_e_ = 8.7 ps and *S*^2^ =0.34, while the experimental values are *τ*_e_ =80 ± 4 ps and *S*^2^ =0.65 ± 0.01. The new force field predicts *τ*_e_ = 79.9 ps and *S*^2^ =0.70, in quantitative agreement with the experimental values and significantly better than the original force field (Table7).

## DISCUSSION

We propose a new approach for force field optimizations which aims at reproducing experimental dynamics characteristics using biomolecular MD simulations, in addition to improved prediction of motionally averaged structural properties available from experiment. As the source of experimental data for dynamics fittings, we use ^13^C NMR spin-lattice relaxation times *T*_1_ of various backbone and sidechain carbon atoms, which allow to selectively determine correlation times of both overall molecular reorientations and intramolecular motions. For relative conformational stability and structural fittings, we use motionally averaged experimental values of NMR ^3^*J* couplings over three bonds. The proline residue and its derivative 4-hydroxyproline with relatively simple structure and sidechain dynamics were chosen for the assessment of the new approach in this work. Initially, the grid search and simplexed MD simulations identified large number of parameter sets which fit equally well experimental *J* couplings. Using the Arrhenius-type exponential relationship between the force constant and the correlation time, the available MD data for a series of different parameter sets were analyzed to determine the value of the force constant that best reproduces experimental timescale of the sidechain dynamics. Verification of the new force-field parameters against NMR *J* couplings and correlation times showed consistent and significant improvements compared to the original force field in reproducing both structural and dynamics properties. These results suggest that matching experimental timescales of motions together with motionally averaged characteristics is a valid and robust approach for force field parameter optimization. Such a comprehensive approach is not restricted to cyclic proline and 4-hydroxyproline residues and can be extended to sidechain structure and dynamics of other amino acid residues, as well as to the protein backbone. In cases more complex than the Pro or Hyp sidechain dynamics, QM methods may also prove successful in providing information regarding the barrier heights of conformational changes, especially when the interpretation of the NMR relaxation data is not straightforward.
