# Supplementary material for: Motional timescale predictions by molecular dynamics simulations: Case study using proline and hydroxyproline sidechain dynamics
Source: Proteins. 2014 Sep 17;82(2):195–215. doi: 10.1002/prot.24350 (PMC4282583; doi:10.1002/prot.24350)
Supplement: Supplementary file 1 — Supplementary Information [file prot0082-0195-SD1.docx]

**Supporting Information**

Motional Timescale Predictions by Molecular Dynamics Simulations: Case Study Using Proline and Hydroxyproline Sidechain Dynamics

Abil E. Aliev,* Martin Kulke, Harmeet S. Khaneja, Vijay Chudasama, Tom D. Sheppard,
Rachel M. Lanigan

Department of Chemistry, University College London, 20 Gordon Street, London WC1H 0AJ, U.K.

* Author for correspondence: [A.E.Aliev@ucl.ac.uk](mailto:A.E.Aliev@ucl.ac.uk)

**Table S1.** The rms deviations for “NMR vs. MD” comparisons for GPGG in water.*^a^*

|  | rms*_d_* (Ǻ) | rms*_Jq1_* (Hz) | rms*_Jq2_* (Hz) | rms*_Je1_* (Hz) | rms*_Je2_* (Hz) |
| --- | --- | --- | --- | --- | --- |
| AMBER99SB | 0.31 | 0.54 | 0.45 | 0.54 | 0.83 |
| 1 | 0.32 | 0.54 | 0.45 | 0.53 | 0.83 |
| 2 | 0.32 | 0.53 | 0.44 | 0.53 | 0.83 |
| 3 | 0.32 | 0.53 | 0.44 | 0.53 | 0.83 |
| 4 | 0.31 | 0.55 | 0.46 | 0.54 | 0.84 |
| 5 | 0.32 | 0.54 | 0.45 | 0.54 | 0.83 |
|  |  |  |  |  |  |
| 6 | 0.36 | 0.51 | 0.41 | 0.52 | 0.82 |
| 7 | 0.34 | 0.52 | 0.43 | 0.52 | 0.82 |
| 8 | 0.32 | 0.53 | 0.43 | 0.53 | 0.83 |
| 9 | 0.33 | 0.53 | 0.43 | 0.53 | 0.83 |
| 10 | 0.33 | 0.53 | 0.45 | 0.54 | 0.83 |
| 11 | 0.32 | 0.53 | 0.45 | 0.53 | 0.83 |
| 12 | 0.32 | 0.53 | 0.45 | 0.53 | 0.83 |
| 13 | 0.34 | 0.52 | 0.43 | 0.52 | 0.83 |
| 14 | 0.33 | 0.53 | 0.42 | 0.53 | 0.83 |
| 15 | 0.32 | 0.53 | 0.43 | 0.53 | 0.83 |
| 16 | 0.33 | 0.52 | 0.42 | 0.53 | 0.83 |
| 17 | 0.34 | 0.52 | 0.43 | 0.53 | 0.83 |
| 18 | 0.34 | 0.53 | 0.44 | 0.53 | 0.83 |
| 19 | 0.34 | 0.51 | 0.41 | 0.52 | 0.82 |
| 20 | 0.33 | 0.52 | 0.41 | 0.54 | 0.83 |
| 21 | 0.33 | 0.52 | 0.42 | 0.54 | 0.83 |
| 22 | 0.34 | 0.52 | 0.42 | 0.52 | 0.83 |
| 23 | 0.33 | 0.53 | 0.42 | 0.53 | 0.83 |
|  |  |  |  |  |  |
| 24 | 0.33 | 0.53 | 0.43 | 0.53 | 0.83 |
| 25 | 0.33 | 0.53 | 0.43 | 0.53 | 0.83 |

*^a^*Shown are the rms deviations between experiment and MD predictions for distances (rms*_d_*), ^3^*J*-couplings using Karplus coefficients derived empirically for ubiquitin (rms*_Je1_*)^[1]^ and flavodoxin (rms*_Je2_*),^[2]^ and ^3^*J*-couplings using Karplus coefficients derived from the B972/EPR-III (rms*_Jq1_*) and B3LYP/EPR-III calculations (rms*_Jq2_*).^[3]^

**Table S2.** Spin-lattice *T*_1_(^13^C) relaxation times (in ms) of GPGG (214 mM in D_2_O) at various temperatures (in Kelvin) at 150.92 MHz.*^a^*

|  | 274.0 K | 283.0 K | 293.0 K | 300.2 K | 308.9 K | 317.6 K | 326.4 K | 335.1 K |
| --- | --- | --- | --- | --- | --- | --- | --- | --- |
| Pro C^α^ | 500 | 603 | 772 | 984 | 1199 | 1406 | 1678 | 1967 |
| Pro C^δ^ | 284 | 359 | 443 | 546 | 668 | 799 | 937 | 1118 |
| Gly-4 C^α^ | 414 | 549 | 709 | 892 | 1108 | 1353 | 1647 | 2023 |
| Gly-3 C^α^ | 284 | 344 | 444 | 546 | 677 | 819 | 979 | 1172 |
| Gly-1 C^α^ | 282 | 339 | 433 | 533 | 658 | 787 | 960 | 1138 |
| Pro C^β^ | 384 | 479 | 605 | 754 | 912 | 1097 | 1289 | 1539 |
| Pro C^γ^ | 459 | 555 | 722 | 905 | 1097 | 1316 | 1490 | 1783 |

*^a^* Uncertainties of *T*_1_(^13^C) measurements were typically within ±2% of measured values.

**Table S3.** ^13^C Chemical shifts and spin-lattice *T*_1_ of GPGG at 298 K (57 mM in D_2_O, ^13^C Larmor frequency 150.92 MHz).

|  | δ_C_ / ppm | *T*_1_ / ms |
| --- | --- | --- |
| Pro C^α^ | 61.60 | 995±6 |
| Pro C^δ^ | 47.65 | 538±3 |
| Gly-4 C^α^ | 43.90 | 918±3 |
| Gly-3 C^α^ | 43.11 | 553±1 |
| Gly-1 C^α^ | 41.19 | 573±5 |
| Pro C^β^ | 30.09 | 745±3 |
| Pro C^γ^ | 25.05 | 898±4 |
| Gly-1 C | 166.78 |  |
| Pro C | 175.40 |  |
| Gly-3 C | 171.59 |  |
| Gly-4 C | 177.24 |  |

**Table S4.** Correlation times τ_c_ and τ_e_ (in ps) determined using *T*_1_ relaxation times measured for C^α^ and C^γ^ of Pro in GPGG (214 mM in D_2_O) at various temperatures (in Kelvin) at 150.92 MHz.*^a^*

| T / K | τ_c_ (ps) | τ_e_ (ps) |
| --- | --- | --- |
| 335.1 | 23.8 | 14.9 |
| 326.4 | 28.5 | 18.0 |
| 317.6 | 33.6 | 18.8 |
| 308.9 | 39.6 | 23.7 |
| 300.2 | 48.8 | 28.2 |
| 293.0 | 63.6 | 33.9 |
| 283.0 | 84.6 | 45.0 |
| 274.0 | 107.1 | 53.8 |

*^a^* Uncertainties in τ_c_ and τ_e_ values were typically within ±0.5 ps of measured values.

**Table S5.** The rms*_J_*_p_ deviations from 700 ns MD simulations of GPGG in water with variations of *V*_3_ (in kJ mol^-1^) and the phase γ_3_ (in degrees). The corresponding value for the original force field AMBER99SB was **0.663 Hz**.

|  | *V*_3_ = 1.0 | *V*_3_ = 2.0 | *V*_3_ = 3.0 | *V*_3_ = 4.0 | *V*_3_ = 5.0 |
| --- | --- | --- | --- | --- | --- |
| γ_3_ = -50º | 0.920 | 1.763 | 2.501 | 3.044 | 3.447 |
| γ_3_ = -40º | 0.820 | 1.467 | 2.154 | 2.735 | 3.167 |
| γ_3_ = -30º | 0.686 | 1.170 | 1.714 | 2.216 | 2.760 |
| γ_3_ = -20º | 0.589 | 0.820 | 1.199 | 1.534 | 1.991 |
| γ_3_ = -10º | 0.552 | 0.549 | 0.647 | 0.870 | 1.049 |
| γ_3_ = 0º | 0.601 | 0.565 | 0.517 | **0.499** | 0.514 |
| γ_3_ = 10º | 0.694 | 0.818 | 0.979 | 1.115 | 1.244 |
| γ_3_ = 20º | 0.851 | 1.171 | 1.491 | 1.760 | 2.048 |
| γ_3_ = 30º | 1.005 | 1.439 | 1.880 | 2.200 | 2.451 |
| γ_3_ = 40º | 1.122 | 1.652 | 2.198 | 2.534 | 2.774 |
| γ_3_ = 50º | 1.258 | 1.897 | 2.389 | 2.714 | 2.909 |

**Table S6.** The population of the C^γ^-endo conformer (*x*_endo_, in %) from 700 ns MD simulations of GPGG in water with variations of *V*_3_ (in kJ mol^-1^) and the phase γ_3_ (in degrees). The corresponding value for the original force field AMBER99SB was 59.0%. The experimental value is **54.3 %**.

|  | *V*_3_ = 1.0 | *V*_3_ = 2.0 | *V*_3_ = 3.0 | *V*_3_ = 4.0 | *V*_3_ = 5.0 |
| --- | --- | --- | --- | --- | --- |
| γ_3_ = -50º | 45.2 | 31.5 | 20.7 | 13.1 | 7.7 |
| γ_3_ = -40º | 47.0 | 36.0 | 25.9 | 17.7 | 11.9 |
| γ_3_ = -30º | 49.9 | 40.6 | 32.4 | 25.3 | 17.8 |
| γ_3_ = -20º | 52.7 | 46.4 | 40.1 | 35.2 | 28.8 |
| γ_3_ = -10º | 55.4 | 52.4 | 49.4 | 45.3 | 42.6 |
| γ_3_ = 0º | 59.1 | 59.3 | 58.5 | **58.2** | 58.6 |
| γ_3_ = 10º | 61.7 | 64.8 | 67.6 | 69.7 | 71.5 |
| γ_3_ = 20º | 64.9 | 70.6 | 75.5 | 79.3 | 83.1 |
| γ_3_ = 30º | 67.7 | 74.8 | 81.2 | 85.6 | 89.0 |
| γ_3_ = 40º | 69.6 | 78.0 | 85.9 | 90.5 | 93.7 |
| γ_3_ = 50º | 71.8 | 81.7 | 88.8 | 93.3 | 95.9 |

**Table S7.** The order parameter 𝒮^2^ from 700 ns MD simulations of GPGG in water with variations of *V*_3_ (in kJ mol^-1^) and the phase γ_3_ (in degrees). The corresponding value for the original force field AMBER99SB was 0.33. The experimental value is **0.27**.

|  | *V*_3_ = 1.0 | *V*_3_ = 2.0 | *V*_3_ = 3.0 | *V*_3_ = 4.0 | *V*_3_ = 5.0 |
| --- | --- | --- | --- | --- | --- |
| γ_3_ = -50º | 0.31 | 0.38 | 0.50 | 0.63 | 0.73 |
| γ_3_ = -40º | 0.30 | 0.34 | 0.43 | 0.54 | 0.65 |
| γ_3_ = -30º | 0.30 | 0.31 | 0.36 | 0.43 | 0.54 |
| γ_3_ = -20º | 0.30 | 0.29 | 0.31 | 0.33 | 0.38 |
| γ_3_ = -10º | 0.30 | 0.29 | 0.28 | 0.28 | 0.28 |
| γ_3_ = 0º | 0.32 | 0.31 | 0.29 | **0.28** | 0.28 |
| γ_3_ = 10º | 0.33 | 0.34 | 0.35 | 0.36 | 0.38 |
| γ_3_ = 20º | 0.35 | 0.39 | 0.44 | 0.48 | 0.54 |
| γ_3_ = 30º | 0.37 | 0.43 | 0.52 | 0.59 | 0.65 |
| γ_3_ = 40º | 0.39 | 0.48 | 0.60 | 0.68 | 0.75 |
| γ_3_ = 50º | 0.41 | 0.53 | 0.65 | 0.74 | 0.80 |

**Table S8.** The τ*_e_* autocorrelation time τ*_e_* (in ps) from 700 ns MD simulations of GPGG in water with variations of *V*_3_ (in kJ mol^-1^) and the phase γ_3_ (in degrees). The corresponding value for the original force field AMBER99SB was 4.26 ps. The experimental value is **29.7 ps**.

|  | *V*_3_ = 1.0 | *V*_3_ = 2.0 | *V*_3_ = 3.0 | *V*_3_ = 4.0 | *V*_3_ = 5.0 |
| --- | --- | --- | --- | --- | --- |
| γ_3_ = -50º | 5.33 | 6.53 | 7.48 | 7.94 | 8.28 |
| γ_3_ = -40º | 5.56 | 7.45 | 9.70 | 11.67 | 13.70 |
| γ_3_ = -30º | 5.89 | 8.45 | 11.99 | 16.83 | 21.16 |
| γ_3_ = -20º | 6.11 | 9.04 | 13.71 | 21.00 | 30.33 |
| γ_3_ = -10º | 6.24 | 9.39 | 15.30 | 23.45 | 38.55 |
| γ_3_ = 0º | 6.09 | 9.47 | 15.27 | **25.01** | 39.39 |
| γ_3_ = 10º | 6.06 | 8.97 | 13.99 | 21.63 | 35.64 |
| γ_3_ = 20º | 5.83 | 8.27 | 11.85 | 17.77 | 25.39 |
| γ_3_ = 30º | 5.48 | 7.28 | 9.32 | 12.64 | 16.41 |
| γ_3_ = 40º | 5.18 | 6.35 | 7.01 | 7.88 | 8.88 |
| γ_3_ = 50º | 4.85 | 5.20 | 5.22 | 5.02 | 4.29 |

**Table S9.** Conformational populations and geometries of three Pro rings in ubiquitin in water as predicted by 1 μs long MD simulations.

| Residue | Parameter set | *P*_exo_ (°) | *P*_endo_ (°) | χ_m_ (°) | *x*_endo_ (%) | *N*^χ2^ | ^3^*J^calc^*(C′-H^α^)*^a,b^* | ^3^*J^calc^*(C′-H^α^) *^a,c^* |
| --- | --- | --- | --- | --- | --- | --- | --- | --- |
| Pro-19 | Amber99SB*-ILDN | 7 | 185 | 35.6 | 47.5 | 67.77 | 1.28 | 1.33 |
|  | 25 | 7 | 185 | 38.8 | 41.2 | 14.41 | 1.26 | 1.29 |
| Pro-37 | Amber99SB*-ILDN | 13 | 178 | 35.6 | 50.3 | 67.27 | 1.37 | 1.50 |
|  | 25 | 12 | 179 | 39.1 | 54.3 | 10.64 | 1.40 | 1.56 |
| Pro-38 | Amber99SB*-ILDN | 1 | 188 | 36.9 | 19.1 | 28.83 | 1.14 | 1.04 |
|  | 25 | 2 | 189 | 39.5 | 13.9 | 4.31 | 1.12 | 1.00 |

*^a^* Experimental values of *J*(C′-H^α^)-couplings were 1.22 (Pro-19), 1.71 (Pro-37) and 1.06 Hz (Pro-38);
*^b^* Calculated using *J* = 3.72 cos^2^(φ + 120) - 2.28 cos(φ + 120) + 1.28)^[1]^; *^c^* Calculated using *J* = 4.32 cos^2^(φ + 115.9) – 1.53 cos(φ + 115.9) + 0.59),^[3]^ derived for Pro residues using B3LYP/EPR-III calculations.

**Table S10.** The rms deviations for “NMR vs. MD” comparisons for ubiquitin ^3^*J*-couplings in water. Parameters of Karplus equations (θ, *A*, *B* and *C*) are also shown.*^a^*

|  | θ / º | *A* / Hz | *B* / Hz | *C* / Hz | Amber99SB*-ILDN | (39) |
| --- | --- | --- | --- | --- | --- | --- |
| ^3^*J*(H^N^-H^α^) | -60 | 9.44 | -1.53 | -0.07 | 0.86 | 0.86 |
|  | -64.51 | 9.14 | -2.28 | -0.29 | 1.01 | 1.00 |
|  | -60 | 7.09 | -1.42 | 1.55 | 0.92 | 0.93 |
|  | -60 | 7.9 | -1.05 | 0.65 | 1.15 | 1.15 |
| ^3^*J*(H^N^-C^β^) | 60 | 5.15 | 0.01 | -0.32 | 1.57 | 1.55 |
|  | 58.18 | 4.58 | -0.36 | -0.31 | 0.89 | 0.88 |
|  | 60 | 3.06 | -0.74 | 0.13 | 0.32 | 0.31 |
|  | 60 | 2.9 | -0.56 | 0.18 | 0.33 | 0.32 |
| ^3^*J*(H^N^-C′) | 180 | 5.58 | -1.06 | -0.3 | 0.49 | 0.49 |
|  | 172.49 | 5.34 | -1.46 | -0.29 | 0.60 | 0.60 |
|  | 180 | 4.29 | -1.01 | 0 | 0.48 | 0.49 |
|  | 180 | 4.41 | -1.36 | 0.24 | 0.54 | 0.54 |
| ^3^*J*(C′-H^α^) | 120 | 4.38 | -1.87 | 0.56 | 0.32 | 0.32 |
|  | 118.61 | 4.77 | -1.85 | 0.49 | 0.36 | 0.34 |
|  | 120 | 3.72 | -2.18 | 1.28 | 0.33 | 0.32 |
|  | 120 | 3.76 | -1.63 | 0.89 | 0.35 | 0.34 |
| ^3^*J*(H^α^-N) | 60 | -0.88 | -0.61 | -0.27 | 0.21 | 0.20 |
| rms_av_*^b^* | - | - | - | - | 0.63 | 0.63 |

*^a^* The first 16 equations for ^3^*J*(H^N^-H^α^), ^3^*J*(H^N^-C^β^), ^3^*J*(H^N^-C′) and ^3^*J*(C′-H^α^) (of the form of *J* = *A* cos^2^(φ + θ) + *B* cos(φ + θ) + *C*) are from Table I of reference [4] (see references therein); the last equation for ^3^*J*(H^α^-N) (of the form of *J* = *A* cos^2^(ψ + θ) + *B* cos(ψ + θ) + *C*) is from reference [5]. *^b^* The rms value averaged over 17 values.

**Table S11.** ^13^C Chemical shifts and spin-lattice *T*_1_ of VAPG at 298 K (77 mM in H_2_O:D_2_O (9:1), ^13^C Larmor frequency 150.92 MHz).

|  | *trans-*VAPG | | *cis-*VAPG | |
| --- | --- | --- | --- | --- |
|  | δ_C_ / ppm | *T*_1_ / ms | δ_C_ / ppm | *T*_1_ / ms |
| Pro C^α^ | 61.21 | 641±6 | 61.45 | 632±10 |
| Val C^α^ | 58.90 | 751±4 | 58.93 |  |
| Ala C^α^ | 48.55 | 614±4 | 48.87 | 558±11 |
| Pro C^δ^ | 48.47 | 375±3 | 48.06 |  |
| Gly C^α^ | 43.85 | 771±2 | 44.18 |  |
| Val C^β^ | 30.62 | 794±2 | 30.57 |  |
| Pro C^β^ | 30.04 | 574±4 | 31.92 |  |
| Pro C^γ^ | 25.21 | 639±4 | 22.48 | 468±18 |
| Val C^γ′^ | 18.22 |  | 18.23 |  |
| Val C^γ′^ | 17.45 |  | 17.46 |  |
| Ala C^β^ | 15.91 |  | 16.34 |  |
| Val C | 169.55 |  | 169.16 |  |
| Ala C | 173.27 |  | 173.71 |  |
| Pro C | 174.23 |  | 173.78 |  |
| Gly C | 176.99 |  | 176.81 |  |

**Table S12.** ^1^H NMR chemical shifts of angiotensin (16 mM solution in D_2_O, 298 K).

| Residue | Proton | δ / ppm |
| --- | --- | --- |
| Asp^1^ | H^α^ | 4.38 |
|  | H^β^ | 2.97 & 3.07 |
| Arg^2^ | H^α^ | 4.38 |
|  | H^β^ | 1.74 |
|  | H^γ^ | 1.49 & 1.56 |
|  | H^δ^ | 3.16 |
| Val^3^ | H^α^ | 4.11 |
|  | H^β^ | 1.99 |
|  | H^γ^ | 0.88 & 0.92 |
| Tyr^4^ | H^α^ | 4.62 |
|  | H^β^ | 2.89 & 2.95 |
|  | H^δ^ | 7.10 |
|  | H^ε^ | 6.76 |
| Ile^5^ | H^α^ | 4.08 |
|  | H^β^ | 1.74 |
|  | H^γ1^ | 1.13 & 1.39 |
|  | H^γ2^ | 0.81 |
|  | H^δ^ | 0.82 |
| His^6^ | H^α^ | 4.86 |
|  | H^β^ | 3.12 & 3.19 |
|  | H^δ2^ | 7.29 |
|  | H^ε1^ | 8.62 |
| Pro^7^ | H^α^ | 4.40 |
|  | H^β3^ | 2.24 |
|  | H^β2^ | 1.87 |
|  | H^γ3^ | 1.99 |
|  | H^γ2^ | 1.99 |
|  | H^δ3^ | 3.80 |
|  | H^δ2^ | 3.57 |
| Phe^8^ | H^α^ | 4.67 |
|  | H^β^ | 3.12 & 3.22 |
|  | H^δ^ | 7.32 |
|  | H^ε^ | 7.38 |
|  | H^ζ^ | 7.31 |

**Table S13.** ^13^C NMR chemical shifts of angiotensin (16 mM solution in D_2_O, 298 K).

| Residue | Carbon | δ / ppm |
| --- | --- | --- |
| Asp^1^ | C^α^ | 50.12 |
|  | C^β^ | 35.79 |
|  | C^γ^ | 169.15 |
|  | C | 173.65 |
| Arg^2^ | C^α^ | 54.05 |
|  | C^β^ | 28.77 |
|  | C^γ^ | 24.83 |
|  | C^δ^ | 41.11 |
|  | C^ζ^ | 157.27 |
|  | C | 173.14 |
| Val^3^ | C^α^ | 59.87 |
|  | C^β^ | 30.99 |
|  | C^γ^ | 18.47 & 18.95 |
|  | C | 173.32 |
| Tyr^4^ | C^α^ | 55.37 |
|  | C^β^ | 36.98 |
|  | C^γ^ | 128.58 |
|  | C^δ^ | 131.13 |
|  | C^ε^ | 115.83 |
|  | C^ζ^ | 155.02 |
|  | C | 172.93 |
| Ile^5^ | C^α^ | 58.33 |
|  | C^β^ | 36.65 |
|  | C^γ1^ | 25.02 |
|  | C^γ2^ | 15.14 |
|  | C^δ^ | 10.39 |
|  | C | 173.15 |
| His^6^ | C^α^ | 51.02 |
|  | C^β^ | 26.33 |
|  | C^γ^ | 128.53 |
|  | C^δ2^ | 118.26 |
|  | C^ε1^ | 115.83 |
|  | C | 170.21 |
| Pro^7^ | C^α^ | 61.04 |
|  | C^β^ | 29.97 |
|  | C^γ^ | 25.16 |
|  | C^δ^ | 48.68 |
|  | C | 174.37 |
| Phe^8^ | C^α^ | 55.00 |
|  | C^β^ | 37.09 |
|  | C^γ^ | 137.11 |
|  | C^δ^ | 129.94 |
|  | C^ε^ | 129.33 |
|  | C^ζ^ | 127.78 |
|  | C | 175.51 |

**Table S14:** Experimental *J*-couplings of the Pro residue of angiotensin (16 mM solution in D_2_O, 298 K) determined using full lineshape analysis. Alternative numbering of protons is also included (as in Figure 2 of reference [6]). The standard deviation is estimated to be ≤ 0.1 Hz.

| Proton Numbering as in ref. [6] | IUPAC  Labelling | *J-*Couplings (Hz) |
| --- | --- | --- |
|  |  | Pro-7 |
| 1-2 | H^α^-H^β3^ | 8.56 |
| 1-3 | H^α^-H^β2^ | 5.54 |
| 2-3 | H^β3^-H^β2^ | -12.98 |
| 2-4 | H^β3^-H^γ3^ | 7.02 |
| 2-5 | H^β3^-H^γ2^ | 7.45 |
| 3-4 | H^β2^-H^γ3^ | 6.80 |
| 3-5 | H^β2^-H^γ2^ | 6.79 |
| 4-5 | H^γ3^-H^γ2^ | -13.46 |
| 4-6 | H^γ3^-H^δ3^ | 6.59 |
| 4-7 | H^γ3^ –H^δ2^ | 6.48 |
| 5-6 | H^γ2^-H^δ3^ | 7.22 |
| 5-7 | H^γ2^-H^δ2^ | 7.08 |
| 6-7 | H^δ3^ -H^δ2^ | 10.17 |

**Table S15.** ^13^C Chemical shifts and spin-lattice *T*_1_ relaxation times of angiotensin at 298 K (16 mM in D_2_O, ^13^C Larmor frequency 150.92 MHz).

|  | δ_C_ / ppm | *T*_1_ / ms |
| --- | --- | --- |
| Pro C^α^ | 61.04 | 372±3 |
| Val C^α^ | 59.87 | 347±3 |
| Ile C^α^ | 58.33 | 324±8 |
| Tyr C^α^ | 55.37 | 310±3 |
| Phe C^α^ | 55.00 | 448±12 |
| Arg C^α^ | 54.05 | 355±1 |
| His C^α^ | 51.02 | 327±1 |
| Asp C^α^ | 50.12 | 520±1 |
| Pro C^δ^ | 48.68 | 233±1 |
| Arg C^δ^ | 41.11 | 320±7 |
| Phe C^β^ | 37.09 | 256±8 |
| Tyr C^β^ | 36.98 | 182±5 |
| Ile C^β^ | 36.65 | 349±17 |
| Asp C^β^ | 35.79 | 313±1 |
| Val C^β^ | 30.99 | 365±8 |
| Pro C^β^ | 29.97 | 329±10 |
| Arg C^β^ | 28.77 | 213±6 |
| His C^β^ | 26.33 | 193±8 |
| Pro C^γ^ | 25.16 | 386±12 |
| Ile C^γ1^ | 25.02 | 253±1 |
| Arg C^γ^ | 24.83 | 287±3 |

**Table S16.** The rms*_J_*_p_ deviations from 600 ns MD simulations of AHM in water with variations of *V*_3_ (in kJ mol^-1^) and the phase γ_3_ (in degrees).

|  | *V*_3_ = 1.3 | *V*_3_ = 2.3 | *V*_3_ = 3.3 | *V*_3_ = 4.3 | *V*_3_ = 5.3 | *V*_3_ = 6.3 |
| --- | --- | --- | --- | --- | --- | --- |
| γ_3_ = 10º | 2.489 | 2.163 | 1.915 | 1.356 | 1.288 | 1.017 |
| γ_3_ = 20º | 2.274 | 1.858 | 1.410 | 1.085 | 0.894 | 0.610 |
| γ_3_ = 25º | 2.192 | 1.656 | 1.307 | 0.889 | 0.715 | 0.593 |
| γ_3_ = 30º | 2.023 | 1.543 | 1.112 | 0.732 | 0.646 | 0.604 |
| γ_3_ = 35º | 2.042 | 1.424 | 0.978 | 0.741 | 0.639 | 0.624 |
| γ_3_ = 40º | 1.972 | 1.336 | 0.903 | 0.721 | 0.656 | 0.673 |
| γ_3_ = 50º | 1.808 | 1.195 | 0.810 | 0.704 | 0.705 | 0.729 |
| γ_3_ = 60º | 1.770 | 1.062 | 0.800 | 0.737 | 0.760 | 0.788 |

**Table S17.** The population of the C^γ^-endo conformer (*x*_endo_, in %) from 600 ns MD simulations of AHM in water with variations of *V*_3_ (in kJ mol^-1^) and the phase γ_3_ (in degrees).

|  | *V*_3_ = 1.3 | *V*_3_ = 2.3 | *V*_3_ = 3.3 | *V*_3_ = 4.3 | *V*_3_ = 5.3 | *V*_3_ = 6.3 |
| --- | --- | --- | --- | --- | --- | --- |
| γ_3_ = 10º | 48.0 | 42.2 | 37.8 | 27.8 | 26.7 | 21.7 |
| γ_3_ = 20º | 44.1 | 36.6 | 28.5 | 22.3 | 18.6 | 10.2 |
| γ_3_ = 25º | 42.6 | 32.8 | 26.4 | 17.9 | 13.7 | 8.8 |
| γ_3_ = 30º | 39.5 | 30.7 | 22.3 | 13.1 | 10.2 | 7.1 |
| γ_3_ = 35º | 39.8 | 28.3 | 19.1 | 13.1 | 7.9 | 6.1 |
| γ_3_ = 40º | 38.5 | 26.4 | 17.0 | 11.8 | 6.8 | 3.8 |
| γ_3_ = 50º | 35.3 | 23.3 | 13.7 | 7.7 | 4.7 | 2.6 |
| γ_3_ = 60º | 34.5 | 19.9 | 12.0 | 6.3 | 3.3 | 1.6 |

**Table S18.** The order parameter 𝒮^2^ from 600 ns MD simulations of AHM in water with variations of *V*_3_ (in kJ mol^-1^) and the phase γ_3_ (in degrees).

|  | *V*_3_ = 1.3 | *V*_3_ = 2.3 | *V*_3_ = 3.3 | *V*_3_ = 4.3 | *V*_3_ = 5.3 | *V*_3_ = 6.3 |
| --- | --- | --- | --- | --- | --- | --- |
| γ_3_ = 10º | 0.28 | 0.29 | 0.31 | 0.41 | 0.40 | 0.47 |
| γ_3_ = 20º | 0.29 | 0.32 | 0.39 | 0.46 | 0.52 | 0.68 |
| γ_3_ = 25º | 0.29 | 0.35 | 0.41 | 0.54 | 0.61 | 0.71 |
| γ_3_ = 30º | 0.31 | 0.37 | 0.47 | 0.63 | **0.68** | 0.70 |
| γ_3_ = 35º | 0.31 | 0.40 | 0.52 | 0.62 | 0.73 | 0.77 |
| γ_3_ = 40º | 0.31 | 0.42 | 0.55 | 0.65 | 0.80 | 0.83 |
| γ_3_ = 50º | 0.34 | 0.46 | 0.62 | 0.74 | 0.80 | 0.86 |
| γ_3_ = 60º | 0.35 | 0.51 | 0.65 | 0.77 | 0.84 | 0.88 |

**Table S19.** The τ*_e_* autocorrelation time τ*_e_* (in ps) from 600 ns MD simulations of AHM in water with variations of *V*_3_ (in kJ mol^-1^) and the phase γ_3_ (in degrees).

|  | *V*_3_ = 1.3 | *V*_3_ = 2.3 | *V*_3_ = 3.3 | *V*_3_ = 4.3 | *V*_3_ = 5.3 | *V*_3_ = 6.3 |
| --- | --- | --- | --- | --- | --- | --- |
| γ_3_ = 10º | 49.9 | 67.0 | 100.5 | 120.8 | 199.5 | 254.1 |
| γ_3_ = 20º | 48.2 | 59.8 | 79.1 | 98.8 | 130.8 | 125.2 |
| γ_3_ = 25º | 45.3 | 59.6 | 71.3 | 80.3 | 104.5 | 114.0 |
| γ_3_ = 30º | 43.9 | 54.6 | 64.3 | 58.7 | **79.3** | 101.5 |
| γ_3_ = 35º | 43.8 | 47.6 | 57.4 | 59.1 | 56.6 | 59.9 |
| γ_3_ = 40º | 40.4 | 47.5 | 45.8 | 48.5 | 25.8 | 33.0 |
| γ_3_ = 50º | 37.1 | 38.6 | 34.3 | 29.3 | 25.8 | 12.8 |
| γ_3_ = 60º | 35.2 | 30.7 | 26.1 | 18.7 | 10.6 | 3.9 |

**Table S20:** Experimental ^1^H NMR chemical shifts of Ace-Hyp-NHMe (AHM) and Ace-Hyp-Gly (AHG) measured for 59 mM solutions in D_2_O at 298 K.

| Proton Numbering as in ref. [6] | IUPAC Labelling | δ_H_ (ppm) | δ_H_ (ppm) |
| --- | --- | --- | --- |
|  |  | AHM | AHG |
| Hyp, 1 | H^α^ | 4.505 | 4.575 |
| Hyp, 2 | H^β3^ | 2.395 | 2.414 |
| Hyp, 3 | H^β2^ | 2.155 | 2.178 |
| Hyp, 5 | H^γ2^ | 4.637 | 4.620 |
| Hyp, 6 | H^δ3^ | 3.720 | 3.693 |
| Hyp, 7 | H^δ2^ | 3.892 | 3.866 |
| COMe | - | 2.201 | 2.170 |
| NMe | - | 2.818 | - |
| Gly | - | - | 4.077 & 4.026 |

**Table S21:** Experimental *J*-couplings of Hyp residues of Ace-Hyp-NHMe (AHM) and Ace-Hyp-Gly (AHG) determined for 59 mM solutions in D_2_O at 298 K using full lineshape analysis. The standard deviation is estimated to be ≤ 0.05 Hz.

| Proton Numbering as in ref. [6] | IUPAC Labelling | *J-*Couplings (Hz) | *J-*Couplings (Hz) |
| --- | --- | --- | --- |
|  |  | AHM | AHG |
| 1-2 | H^α^-H^β3^ | 7.89 | 7.98 |
| 1-3 | H^α^-H^β2^ | 8.97 | 8.77 |
| 2-5 | H^β3^-H^γ3^ | 2.52 | 2.71 |
| 3-5 | H^β2^-H^γ3^ | 4.53 | 4.54 |
| 5-6 | H^γ3^-H^γ2^ | 1.87 | 2.01 |
| 5-7 | H^γ3^-H^δ3^ | 4.08 | 4.14 |
| 2-3 | H^β3^-H^β2^ | -13.75 | -13.74 |
| 6-7 | H^δ3^-H^δ2^ | -11.74 | -11.71 |
| 2-6 | H^β3^-H^δ3^ | 1.87 | 1.78 |

**Table S22.** ^13^C Chemical shifts and spin-lattice relaxation times of Ace-Hyp-NHMe (AHM) and Ace-Hyp-Gly (AHG) measured for 59 mM solutions in D_2_O at 298 K (^13^C Larmor frequency 150.92 MHz).

| AHM carbons | AHM  δ_C_ / ppm | AHM  *T*_1_ / ms | AHG carbons | AHG  δ_C_ / ppm | AHG  *T*_1_ / ms |
| --- | --- | --- | --- | --- | --- |
| Hyp C^γ^ | 70.24 | 1572±6 | Hyp C^γ^ | 70.23 | 1247±6 |
| Hyp C^α^ | 59.6 | 1428±11 | Hyp C^α^ | 59.31 | 1095±12 |
| Hyp C^δ^ | 56.78 | 731±13 | Hyp C^δ^ | 56.72 | 545±11 |
| Hyp C^β^ | 38.29 | 811±10 | Hyp C^β^ | 38.29 | 639±9 |
| NCH_3_ | 26.51 |  | Gly C^α^ | 41.66 | 767±7 |
| Ac CH_3_ | 21.35 |  | Ac CH_3_ | 22.17 |  |
| Ac C | 174.1 |  | Ac C | 174.05 |  |
| Hyp C | 175.06 |  | Hyp C | 175.11 |  |

**Figure Captions**

**Figure S1**. Population of the endo ring conformation of NAcPro (*x*^endo^, in %) against the length of the MD simulation (in ns). The expansion of the region between 0-50 ns is also shown.

**Figure S2.** The *E*_dih_(χ_2_) graphs for the χ_2_=CT-CT-CT-CT torsion calculated using Eq. (5) and values of *V*_1_, *V*_2_, *V*_3_ and γ_n_ from Table I.

**Figure S3**. Plot of *ln* (*N*^χ2^) vs. χ_m_ (in degrees) showing a linear dependence with *ln* (*N*^χ2^) = -0.8857 χ_m_ + 36.913 (*r*^2^ = 0.9657).

**Figure S4**. Plot of χ_m_ (in degrees) vs. *V*_3_ (in kJ mol^-1^) showing a linear dependence with χ_m_ = 0.5544 *V*_3_ + 36.268 (*r*^2^ = 0.9785).

**Figure S5**. The overlaid C^γ^-endo and C^γ^-exo conformations of NAcPro, which were used to determine the jump angle Δθ for the C^γ^-H bond directions as a result of the pyrrolidine ring interconversion.

**Figure S6**. Plot of *ln* (τ_e_) (in ps) vs. *V*_3_ (in kJ mol^-1^) showing a linear dependence with *V_3_* (in kJ mol^-1^) = 1.9272 *ln* τ_e_ (in ps) – 2.1881 (with *r*^2^ = 0.9975).

**Figure S7.** Internal correlation function (black line) for the C_γ_-H_γ1_ bond reorientations in Pro-2 of GPGG as a result of the pyrrolidine ring interconversion, as predicted by MD simulations using parameter set (25). The exponential fit ($C\left( t \right)=\mathcal{S}^{2}+\left( 1-\mathcal{S}^{2} \right) e^{-t/\tau_{e}})$using the first 20 ns of the correlation function is shown in red. For clarity, the expanded region of 0 – 3 ns is shown in this figure. Judging by the quality of the fit, a single exponential fit reproduces sufficiently well the internal correlation function. Thus, contributions from other motions (if any) are negligibly small and can be safely disregarded.

**Figure S8**. The sequence of amino acid residues in angiotensin.

**Figure S9**. Fitted (black) and experimental (red, 16 mM in D_2_O, 298 K, 600 MHz) ^1^H NMR multiplets due to seven protons of Pro-7 in angiotensin. Protons are numbered as 1-7: 1= H^α^, 2= H^β3^, 3= H^β2^, 4= H^γ3^, 5= H^γ2^, 6= H^δ3^ and 7= H^δ2^.

**
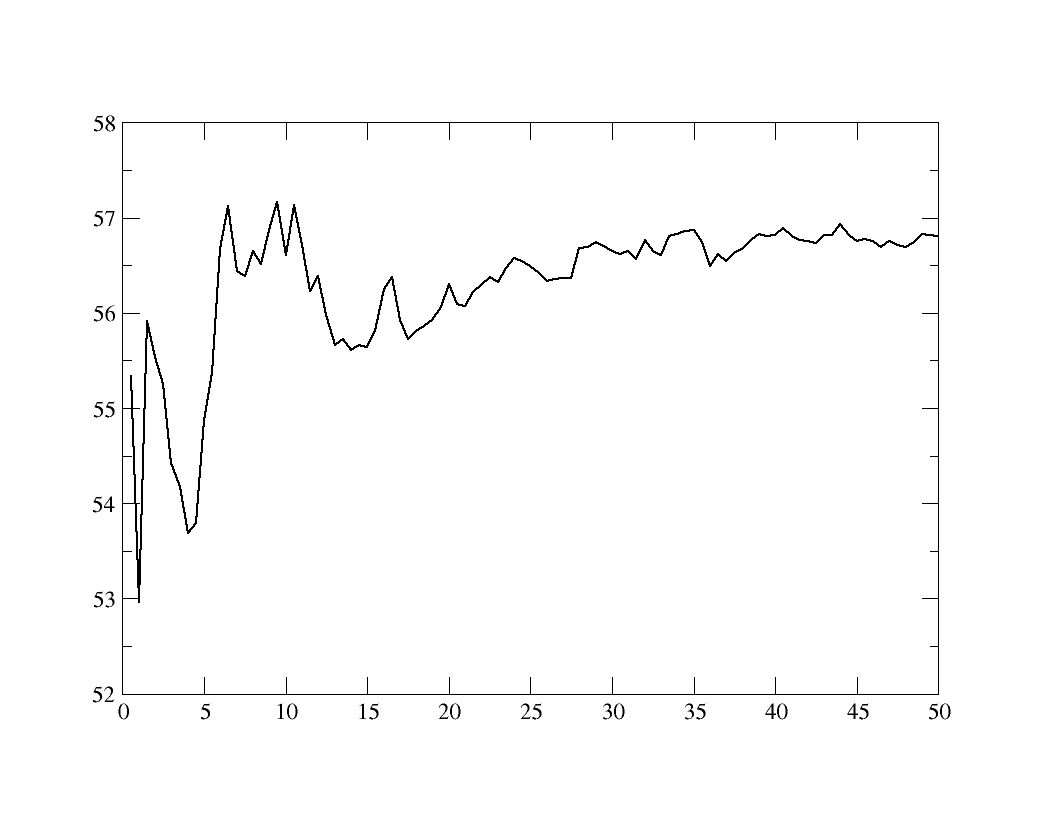

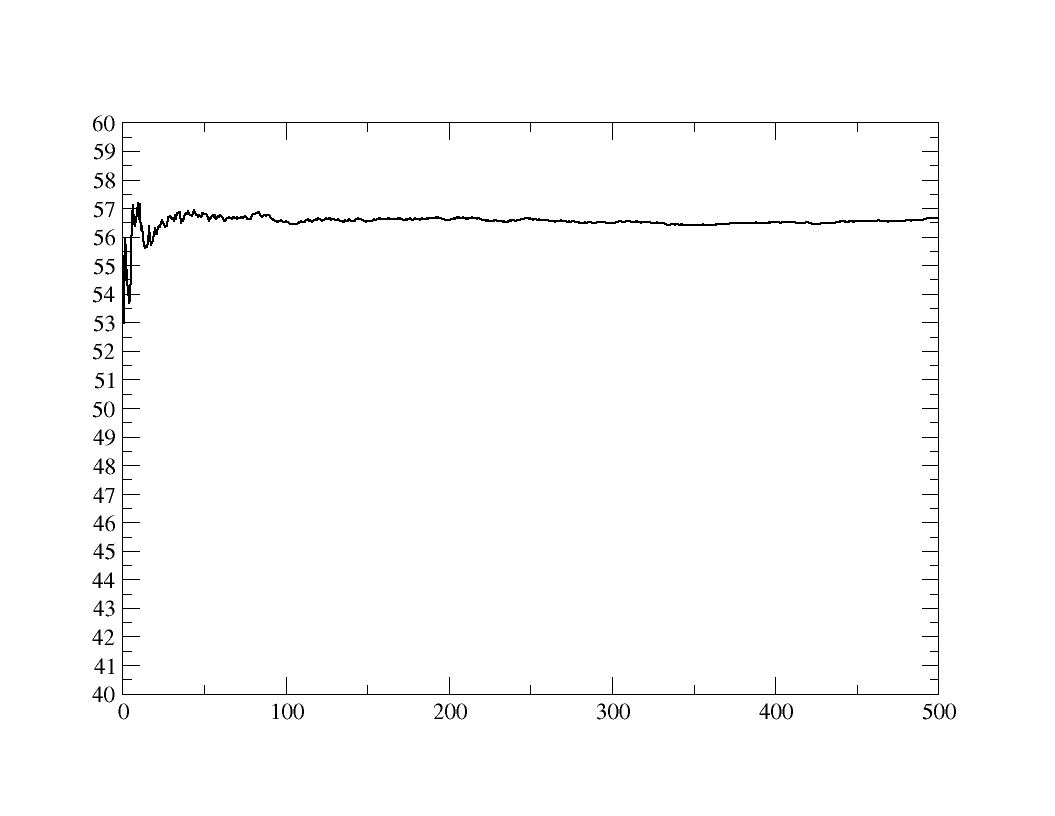
**

Population, *x*^endo^ (%)

Time (ns)

**Figure S1.**

**
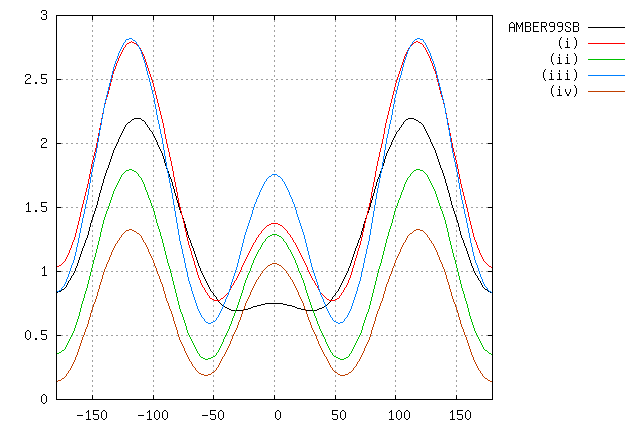
**

Amber99SB

(1)

(2)

(3)

(4)

χ_2_ = CT-CT-CT-CT (º)

*E*_dih_ (kJ mol^-1^)

**Figure S2.**

**Figure S3.**

**Figure S4.**


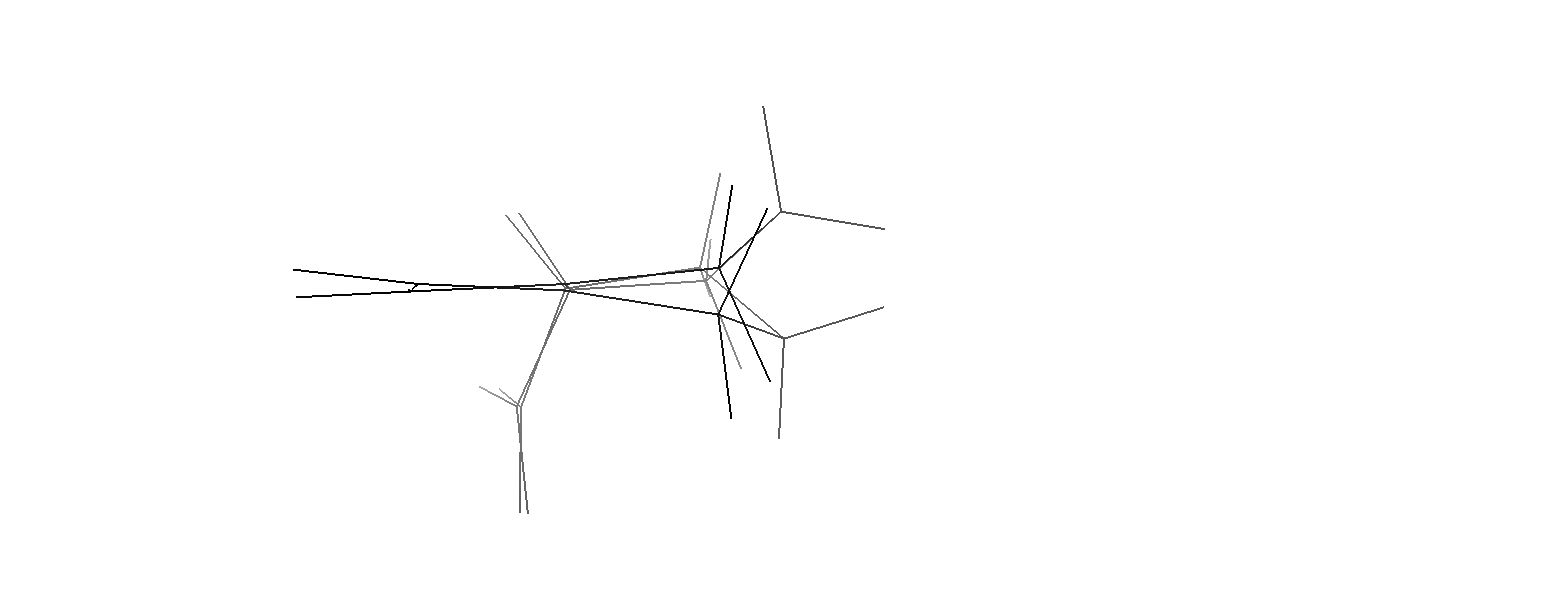


**Figure S5.**

C^γ^-endo

C^γ^-exo

**Figure S6.**


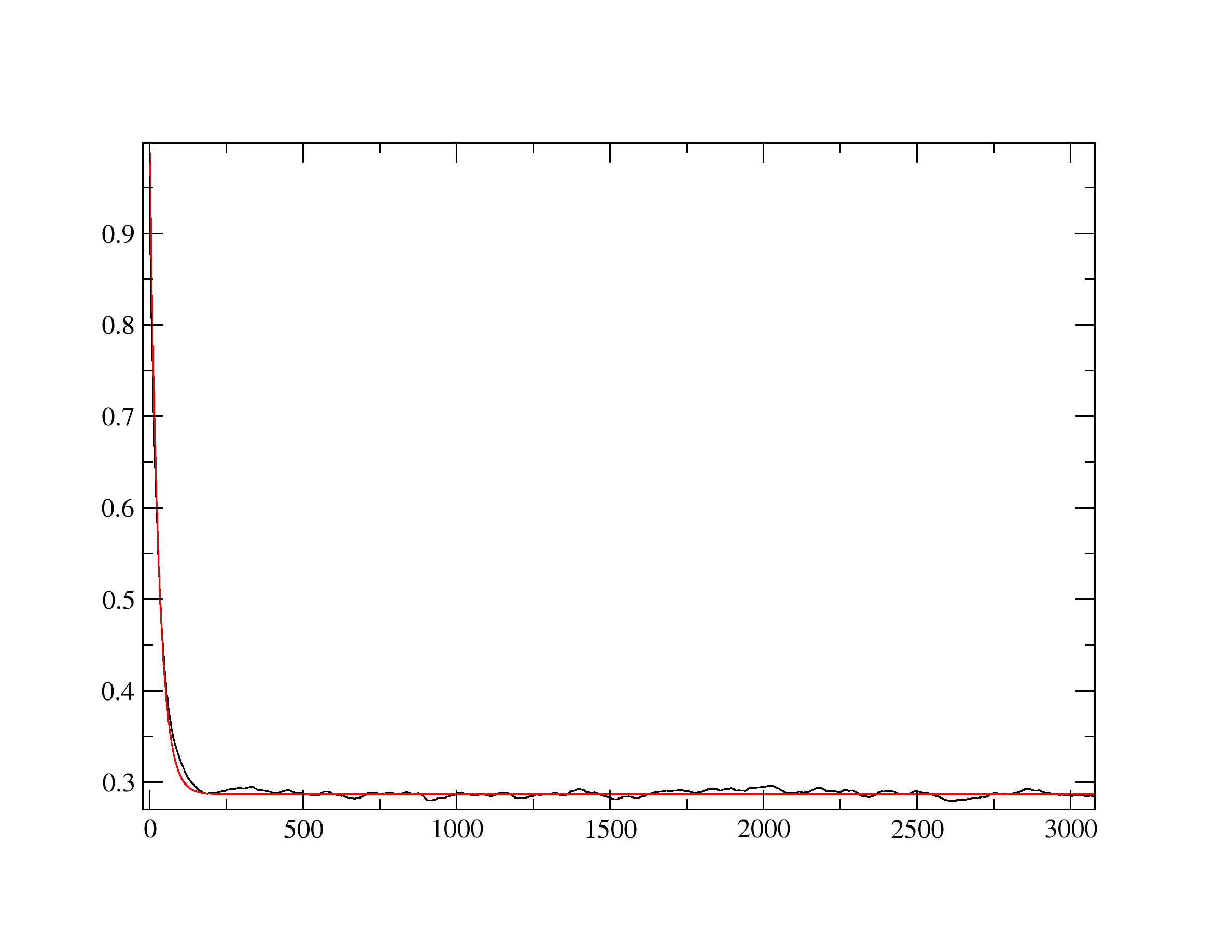


**Figure S7.**

**Figure S8**.


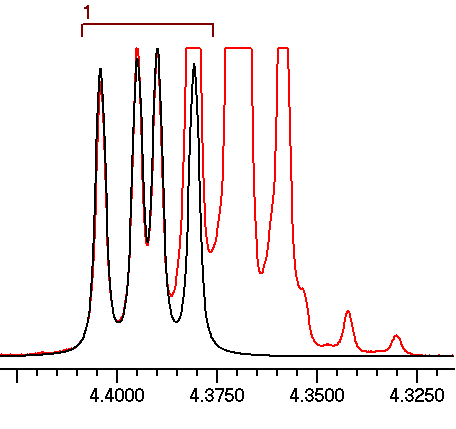

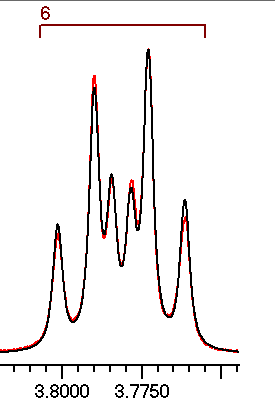

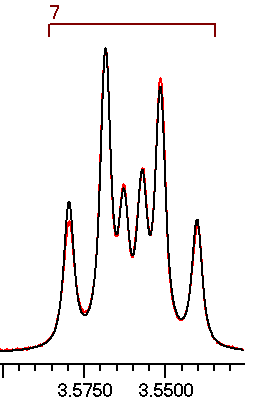

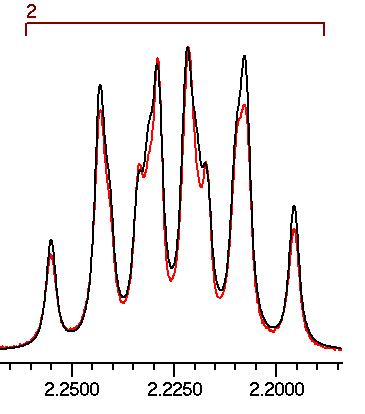

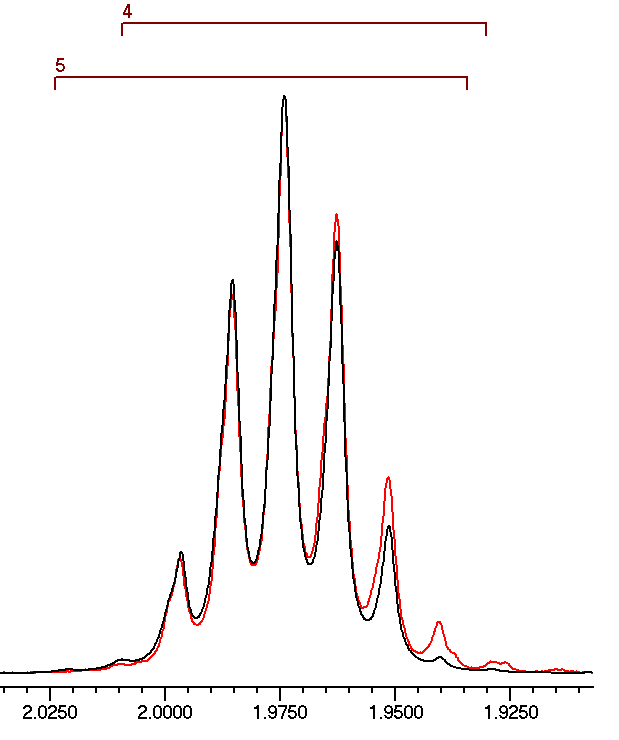

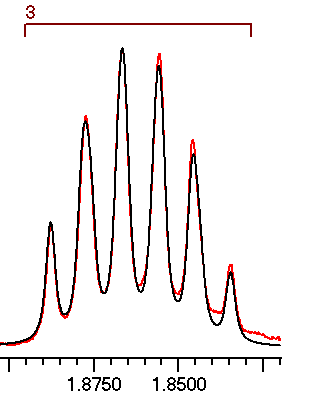


**Figure S9**.

**Synthesis of hydroxyproline peptides.**

All reagents were purchased from Aldrich or AlfaAesar and were used as received without further purification. All reactions were monitored by thin-layer chromatography (TLC) on pre-coated silica gel plates (254 μm). Flash column chromatography was carried out with Kiesegel 60M 0.04/0.063 mm (200-400 mesh) silica gel. Mass spectra were obtained on a VG70-SE mass spectrometer. Melting points were measured with a Gallenkamp apparatus and are uncorrected. Infrared spectra were obtained on a Perkin Elmer Spectrum 100 FTIR Spectrometer operating in ATR mode. Details of NMR measurements are included in Experimental in the main text.

**(2*S*,4*R*)-1-Acetyl-4-hydroxy-*N*-methylpyrrolidine-2-carboxamide (Ace-Hyp-NHMe, AHM)**

To a solution of (2*S*,4*R*)-*N*-Acetyl-4-hydroxyproline (100 mg, 0.58 mmol) in MeOH (4 mL) was added acetyl chloride (0.04 mL, 0.44 mmol) and the reaction mixture heated under reflux for 16 h. After this time, the volatile materials were removed *in vacuo* to afford crude methyl ester (2*S*,4*R*)-methyl 1-acetyl-4-hydroxypyrrolidine-2-carboxylate. Purification by flash column chromatography (5-10% MeOH/CH_2_Cl_2_) gave (2*S*,4*R*)-methyl 1-acetyl-4-hydroxypyrrolidine-2-carboxylate as a colourless oil (107 mg, 0.57 mmol, 99%).^[7]^ (2*S*,4*R*)-Methyl 1-acetyl-4-hydroxypyrrolidine-2-carboxylate (107 mg, 0.57 mmol) was dissolved in saturated methanolic methylamine solution (2 mL) and the reaction mixture stirred for 16 h at room temperature. After this time, the volatile materials were removed *in vacuo* to afford crude (2*S*,4*R*)-1-acetyl-4-hydroxy-*N*-methylpyrrolidine-2-carboxamide. Purification by flash column chromatography (5-20% MeOH/CH_2_Cl_2_) gave (2*S*,4*R*)-1-acetyl-4-hydroxy-*N*-methylpyrrolidine-2-carboxamide as a white solid (75 mg, 0.40 mmol, 69%). m.p. 164-167 °C (*lit. m.p*. 165 °C)^[8]^; IR (solid) 3471, 3304, 3158, 2950, 2918, 1670, 1625, 1538 cm^-1^; LRMS (ES^+^) 187 (100, [M+H]^+^), 180 (40), 174 (35); ^1^H and ^13^C NMR data are included in Tables S20-S22.

HRMS (ES^+^) calcd for C_8_H_15_N_2_O_3_ [M+H]^+^ 187.1083, observed 187.1084.

**2-((2*S*,4*R*)-1-Acetyl-4-hydroxypyrrolidine-2-carboxamido)acetic acid (Ace-Hyp-Gly, AHG)**

To a biphasic solution of (2*S*,4*R*)-*N*-Acetyl-4-hydroxyproline (510 mg, 2.95 mmol) and Glycine *tert‑*butyl ester (410 μL, 393 mg, 3.0 mmol) in CH_2_Cl_2_ (25 mL) and H_2_O (25 mL) was added EDC (1.36 g, 7.1 mmol) and HOBt (380 mg, 2.8 mmol) and the reaction mixture stirred for 60 h at room temperature. After this time, the CH_2_Cl_2_ layer was separated and the aqueous layer washed with CHCl_3_ (3 × 25 mL). The combined organic layers were washed with 1M NaHCO_3_ (25 mL), 1M HCl (25 mL) and saturated NaCl (25 mL). The organic phase was dried (MgSO_4_) and the solvents removed *in vacuo* to afford crude *tert*-butyl 2-((2*S*,4*R*)-1-acetyl-4-hydroxypyrrolidine-2-carboxamido)acetate as an orange solid. The crude *tert*-butyl 2-((2*S*,4*R*)-1-acetyl-4-hydroxypyrrolidine-2-carboxamido)acetate was dissolved in CH_2_Cl_2_ (10 mL), TFA (10 mL) was added to the solution and the reaction mixture stirred at room temperature for 6 h. After this time, the volatile materials were removed *in vacuo* to afford 2-((2*S*,4*R*)-1-Acetyl-4-hydroxypyrrolidine-2-carboxamido)acetic acid as a white solid (322 mg, 1.40 mmol, 47%). m.p. 180-182 °C; IR (solid) 3465, 3297, 2956, 2925, 2854, 1713, 1642, 1610, 1545 cm^-1^; LRMS (ESˉ); 229 (100, [M-Hˉ); HRMS (ESˉ) calcd for C_9_H_13_N_2_O_5_ [M-H]ˉ 229.0824, observed 229.0818. ^1^H and ^13^C NMR data are included in Tables S20-S22.

**References**

1. Hu, J.-S.; Bax, A. *J. Am. Chem. Soc.* **1997**, *119*, 6360.
2. Schmidt, J. M.; Blümel, M.; Löhr, F.; Rüterjans, H. *J. Biomol. NMR* **1999**, *14*, 1.
3. Aliev, A.E.; Courtier-Murias, D. *J. Phys. Chem. B* **2010**, *114*, 12358.
4. Case*,* D. A.; Scheurer*,* C.; Brüschweiler*,* R. *J. Am. Chem. Soc.* **2000**, *122*, 10390*.*
5. Wang, A.C.; Bax, A. *J. Am. Chem. Soc.* **1995**, *117*, 1810.
6. Aliev, A.E.; Courtier-Murias, D. J. Phys. Chem. B **2007**, ***111***, 14034.
7. Kuemin, M.; Nagel, Y. A.; Schweizer, S.; Monnard, F. W.; Ochsenfeld, C.; Wennemers, H. *Angew. Chem.*, **2010**, *36*, 6468.
8. Smolikova, J.; Vitek A.; Blaha, K. *Coll. Czechoslov. Chem. Commun.*, **1971**, *36*, 2474.
